# Institutional factors influencing vaccine access in Canada: a scoping review

**DOI:** 10.1186/s12889-026-27593-w

**Published:** 2026-04-29

**Authors:** Subrana Rahman, Charlotte Zhang, Anaam Khan, Andrea Bowra, Christopher Adanty, David Sanchez Villa, Gil Maor, Kristy Scarfone, Jillian Kohler

**Affiliations:** 1 Leslie Dan Faculty of Pharmacy, University of Toronto, Toronto, ON Canada; 2 Munk School of Global Affairs and Public Policy, Univeristy of Toronto, Toronto, Canada; 3 Dalla Lana School of Public Health, University of Toronto, Toronto, ON Canada

**Keywords:** Vaccines, Vaccine access, Canada, Scoping Review, Institutional Determinants, Health Equity, Health Systems Strengthening

## Abstract

**Background:**

While existing literature has examined vaccine access primarily at individual and community levels in Canada, there remains limited synthesis and knowledge consolidation of how institutional factors shape vaccine access. Where institutional influences have been explored, prior reviews have been limited in scope to specific vaccine programs, populations, or single jurisdictions. This scoping review addresses this gap by systematically mapping how formal and informal institutional factors across federal and provincial governance structures shape vaccine access among all vaccine types and populations in Canada.

**Methods:**

The review adhered to PRISMA Extension for Scoping Reviews (PRISMA-ScR) and Joanna Briggs Institute (JBI) methodological frameworks. Database searches identified English-language sources published 2004–2024. Screening and data extraction were conducted through Zotero, Covidence, and Microsoft Excel. Included sources (*n* = 134) underwent iterative inductive-deductive thematic coding. Finally, descriptive analysis synthesized findings into narrative summaries categorizing institutional factors as barriers or facilitators.

**Results:**

Six institutional barriers in Canada to vaccine access were identified: inconsistent vaccine supply and availability, public funding and coordination structures, geographic constraints, regulatory prioritization mechanisms, vaccine hesitancy, and information access inequities. Four facilitators demonstrated effectiveness: expanded vaccinator scope of practice, technological advancements, targeted vaccination initiatives, and inter-institutional and community collaboration.

**Conclusions:**

This review extends current evidence on factors influencing vaccine access through consolidation of evidence on institutional determinants of vaccine access in Canada across disciplines, governance levels, and vaccine types with an explicit application of North’s institutional theory framework. Findings support evidence-based policy development and health systems strengthening to advance vaccine equity. Priority interventions include harmonization of cross-jurisdictional frameworks, sustainable funding models for community-based delivery, and enhanced data infrastructure supporting equitable access.

**Supplementary Information:**

The online version contains supplementary material available at 10.1186/s12889-026-27593-w.

## Background

Vaccines are an essential public health tool with demonstrated efficacy in addressing a wide range of life-threatening diseases and conditions globally [1]. In Canada, vaccines have successfully eliminated, contained, and controlled previously common diseases such as smallpox, measles and polio [2]. However, despite Canada’s universal healthcare system, substantial geographic and demographic disparities persist in vaccine availability, accessibility, and acceptability. These three dimensions constitute the foundational framework of vaccine equity, defined as the state in which all individuals, regardless of socioeconomic status, race, ethnicity, geographic location, or other social determinants, possess equitable opportunity for vaccination [3].

Vaccine access is determined by interconnected institutional-level influences, including policies and collective norms that govern vaccine procurement, distribution and delivery [4]. To analyze these influences systematically, we draw on Douglas North’s institutional theory, which defines institutions as humanly devised constraints that structure political, economic, and social interaction [4]. Within North’s framework, institutions are further distinguished into formal institutions (e.g., laws or rights) and informal institutions (e.g., social norms and cultural practices) that enable or constrain individual and collective actions at the systems level [4]. North’s framework has been widely applied in political economy and governance research to examine how institutional design shapes policy outcomes and resource distribution, but has seen limited application to public health and vaccine access specifically [5, 6]. North’s typology, however, is well suited to the Canadian immunization context, where formal institutions interact with informal institutions to produce observable patterns in vaccine access.

There is a wealth of literature that has examined vaccine hesitancy, confidence, and various barriers and facilitators to uptake at individual and community levels in Canada and globally [7–9]. Within the COVID-19 pandemic context, researchers have identified system-level determinants of vaccine access and uptake [7], while studies on trust in health authorities, misinformation, and political views have expanded understanding of hesitancy-specific determinants [8, 9]. However, prior Canadian reviews have typically been constrained in scope, focusing on specific vaccine programs such as adult vaccination [10, 11], particular populations such as marginalized communities [9], COVID-19 vaccination only [12, 13], or single provincial jurisdictions [14]. While these contributions provide valuable program- and population-specific insights, many of the institutional barriers have been identified repeatedly across different studies over the past two decades. The evidence, however, remains isolated to specific cases and populations, and no existing review has systematically examined whether these barriers represent persistent, cross-cutting patterns or isolated findings that are context-specific.

Our scoping review aims to address this gap by systematically identifying and synthesizing the institutional determinants of vaccine access across all vaccine types, populations and governance levels in Canada. Since scoping reviews are well suited to mapping heterogeneous evidence across multiple contexts, our review utilizes this methodology to consolidate current evidence and identify which institutional barriers persist across contexts and populations. Guided by North’s institutional typology, we examine how formal institutions, including legislation, funding mechanisms, and regulatory frameworks, interact with informal institutions, such as cultural norms and systemic discrimination, to shape vaccine access [4]. By situating our analysis at the systems level, our findings can comprehensively identify where institutional interventions can most effectively advance vaccine equity in Canada.

## Methods

### Protocol

This scoping review was conducted according to the guidelines for Preferred Reporting Items for Systematic Reviews and Meta Analyses extension for Scoping Reviews (PRISMA-ScR) and Joanna Briggs Institute (JBI) Framework. The Protocol was registered in Open Science Framework (OSF) on January 17, 2025, and updated on April 1st, 2025 (https://osf.io/jd2ge).

### Eligibility criteria

Documents were included in this scoping review based on the following inclusion criteria:

1. Included individuals and communities in Canada, including specific demographic groups such as immigrants, Indigenous populations, and low-resource communities.
2. Examined institutional factors as defined by North [15] that influence vaccine access at the systems level, including formal rules (legislation, policies, funding mechanisms, regulatory frameworks) and informal constraints (organizational norms, professional cultures, systemic discriminations).
3. Assessed the availability, accessibility, acceptability, and uptake of vaccines in Canada.
4. Employed qualitative, quantitative, or mixed methods approaches, as well as systematic reviews and policy analyses methodologies.
5. Published within the last 10–20 years to ensure the inclusion of recent developments in vaccine policies and distribution frameworks.

For academic literature, we excluded studies if they did not address access-related or institutional factors, were conducted outside of Canada, or were not peer-reviewed (e.g. commentaries, abstracts, news articles, and editorials).

Information Sources and Search Strategies.

Both peer-reviewed and grey literature sources were included in this scoping review. Search terms included variations relating to vaccines, access, and availability and filters were applied to identify Canada-specific content. Peer-reviewed literature searches were conducted in OVID Medline, OVID Embase, and Scopus. Searches were designed in collaboration with a health sciences librarian from the University of Toronto. The searches were limited to articles published in English. Sources published in French were excluded due to resource and translation constraints, which may have underrepresented literature from Quebec and non-English-speaking communities. The search timeframe was limited between 2004 and 2024. The beginning of the timeframe aligns with the establishment of the Public Health Agency of Canada (PHAC) in 2004 following the SARS outbreak, which restructured federal oversight of immunization and infectious disease preparedness [16], as well as the Human Genome Project in 2004, which provides a historical marker for exploring how biotechnological innovation, regulatory transformation, and global health governance intersected to shape institutional practices around vaccine access in Canada [17]. The timeframe extends through the COVID-19 pandemic to capture recent developments and two decades of institutional shifts in vaccine governance and infrastructure. Searches were conducted on January 7th, 2025. A test set of articles were identified and compared against initial search results to ensure relevant research articles were captured, increasing search strategy validity. Full peer-reviewed and grey literature search strategies can be found in Appendix A.

Grey literature searches were conducted across all national, provincial, and territorial health department websites as well as through Google search engine’s advanced searching function. Searches for grey literature were limited to publications released after 2014 (10 years) to prioritize currency and policy relevance, as government reports, guidelines, and program documents are frequently superseded by updated versions and may no longer reflect current institutional arrangements. The team initially attempted a 20-year search across government sources, which yielded an unmanageable volume of documents, a substantial proportion of which described programs no longer in operation. Therefore, the team finalized the decision to narrow the search to publications released after 2014. Searches were conducted between January 15th -February 25th, 2025, and were guided by the Joanna Briggs Institute framework [18]. Systematic searches were conducted reviewing the first 10 pages from each query. Additionally, targeted searches for relevant government agencies and organizations that oversee vaccine programs for specific populations were led by DSV with assistance from KS. These searches focused on Indigenous communities, veterans, and refugees (see Appendix B for full list of websites). These populations were selected because each is served by additional dedicated institutions that warrant more specific examination of the distinct institutional barriers that arises. Immunization for Indigenous communities is supported by Indigenous Services Canada (ISC) and run through community-based health centres [19]. Veterans receive additional coverage through Veterans Affairs Canada’s Treatment Benefits program [20]. Refugees access immunization through temporary coverage under the Interim Federal Health Program [21]. While other populations such as people who are unhoused, incarcerated populations and people who use drugs also experience institutional barriers specific to their context, they do not have dedicated vaccination mandates and thus are captured through the broader searches. However, the absence of targeted searches for these populations may have resulted in underrepresentation of literature unique to their vaccine access experiences, which could limit the transferability of our findings specific to these groups.

### Data screening

Identified literature was exported into referencing management software, Zotero, where an initial round of duplicates was removed. References were then imported into Covidence review software for screening using existing eligibility criteria and a second round of duplicates was removed. Title and abstract screening were conducted by three independent reviewers (SR, AK, and CA). The first 10 studies were screened and discussed by all three reviewers for consistency. Conflicts were resolved by two to three reviewers. Full texts were then screened by five independent reviewers (SR, AK, CA, DSV, and GM). Conflicts at this stage were resolved by a minimum of two reviewers. Grey literature sources were assessed for relevance and credibility based on authoring organization, publication date, and alignment with the review’s focus on institutional factors. Documents published by federal and provincial government agencies and established health organizations were prioritized, while sources lacking clear authorship or institutional affiliation were excluded. The choice of source authority and relevance as quality indicators is consistent with JBI guidance for grey literature appraisal in scoping reviews [18].

### Data extraction and synthesis

To operationalize North’s institutional framework in data extraction and synthesis, we first consider regulatory decisions by governmental agencies that define vaccine development, procurement, and distribution as formal institutions [22, 23]. Informal institutions include systemic and cultural norms that define how physicians deliver, and recipients perceive and experience vaccines at the point-of-care [24]. We further distinguish institutional determinants from individual and community-level factors using the following definitions: Individual factors refer to personal characteristics and preferences, while community factors refer to local interpersonal dynamics independent of governance structures as defined by Bronfenbrenner in the Social Ecological Model [25]. We classify a factor as institutional when it originates from formal policy decisions or informal systemic norms at the systems level. For example, vaccine hesitancy driven by personal concerns constitutes an individual-level factor, whereas vaccine hesitancy as a rational response to prolonged systemic discrimination reflects an institutional determinant [26]. We further distinguish institutional determinants from the broader category of social determinants of health, which are upstream social conditions such as income, education, and housing that influence health outcomes [27].

While institutional factors and social determinants often interact, our analysis focuses on governance decisions within vaccine programs. Furthermore, to examine the equity implications of our identified institutional factors and how they distribute across populations, we applied the PROGRESS-Plus equity framework in the analysis of our findings [28]. PROGRESS-Plus categorizes characteristics associated with health inequities, including place of residence, race/ethnicity, occupation, gender, education, socioeconomic status, and social capital, alongside context-specific vulnerability markers such as housing instability or incarceration [28].

Data extraction was conducted in Excel (version 16.89.1) and included the following descriptive parameters: title, authors, journal, digital object identifier (DOI), study type, vaccine, population, age group, and geographic location. Overall, an inductive-deductive hybrid approach was taken in data analysis. Pre-determined codes were first used for inductive analysis listed in Table 1. Four coders conducted the inductive coding process. In the initial calibration phase, the full research team met to collaboratively analyze the first iteration of codes. The first five articles were coded collectively to reach consensus on the analytical approach. Deductive codes were then created using recurrent themes based on initial literature searching as well as author content expertise. The coding framework was then refined by combining and separating deduced themes throughout the initial process.

Following this calibration phase, articles were distributed among team members in larger sections for independent coding. Data extraction followed the PRISMA extension for scoping reviews checklist and was conducted by four independent reviewers (SR, AK, CA, and DSV). To track workflow, the team developed an organizational system to monitor coding completion, progress, and articles requiring further team deliberation. Articles were not cross coded by a second researcher. However, when disagreements or ambiguities arose, the relevant article was circulated to additional team members, and consensus was reached through group discussion in scheduled meetings. Resulting data was summarized in a cleaned extraction table, and descriptive figures were created using R (v. 4.5.1) with *ggplot2* and *sf* packages.

**Table 1:** Pre-determined themes included in the data extraction template [4]

Theme	Definition	Application to North’s Institutional Framework
Healthcare System Structures	How healthcare services are organized and delivered, including vaccination programs and policies.	Formal institutional factor
School Based Programs	Vaccine delivery and programming in school settings.	Formal institutional factor
Publicly Funded Vaccine	Vaccines funded or subsidized by government agencies.	Formal institutional factor
Cultural Norms and Beliefs	Shared understandings and expectations, cultural norms within healthcare institutions and the broader society (e.g. racism, sexism, etc.)	Informal institutional factor
Digital and Administrative Systems	Systems for the management and operational processes of healthcare organizations. The integration of digital technologies and information.	Formal institutional factor
Access to Information	Information systems: Mechanisms for sharing vaccine-related information and coordinating vaccination efforts.	Informal institutional factor
Vaccinator Qualification	Expanding the role of a professional/non-professional to administer vaccines	Formal institutional factor
Role of Pharmacists	Expanding the role of pharmacists to administer vaccines	Formal institutional factor

Thematic inductive analysis was conducted on the extracted data, adding the following sub-themes to further characterize the data: inter-institutional collaboration, regulatory prioritization for vaccines, location of vaccination, and the role of community members and non-professionals. Finally, a descriptive analysis was undertaken to provide a summary of the included material. Results are presented in the following section as a narrative summary.

## Results

Database searches identified 3,147 articles. After duplicates were removed, the titles and abstracts of 2,477 studies were screened and 2,021 studies were excluded. The full texts of the remaining 456 studies were screened, and an additional 322 studies were excluded leaving 134 peer-reviewed studies to be included in this review. A summary of all included articles (*n* = 134), including study characteristics, institutional factors, and key findings, is provided in Appendix C.

**Fig. 1:**
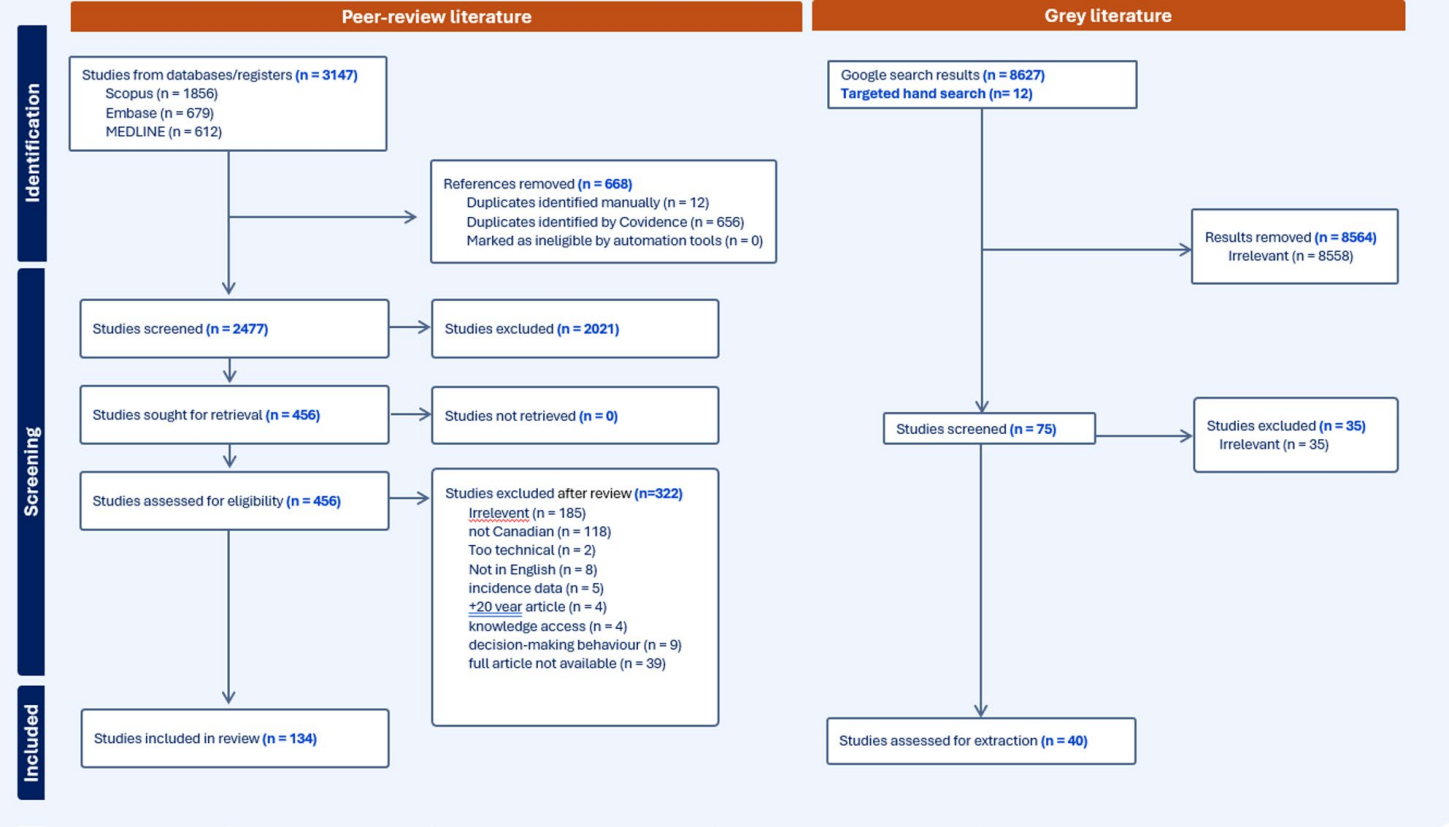
PRISMA flow diagram illustrating the study selection process. Figure generated using Covidence systematic review software

An additional 34 grey literature documents were identified through Google searches, producing a total of 169 documents included in this review. In keeping with the PRISMA extension for scoping review guidelines, the full search and screening process is provided as a PRISMA Flow Chart in Fig. 1.

**Fig. 2:**
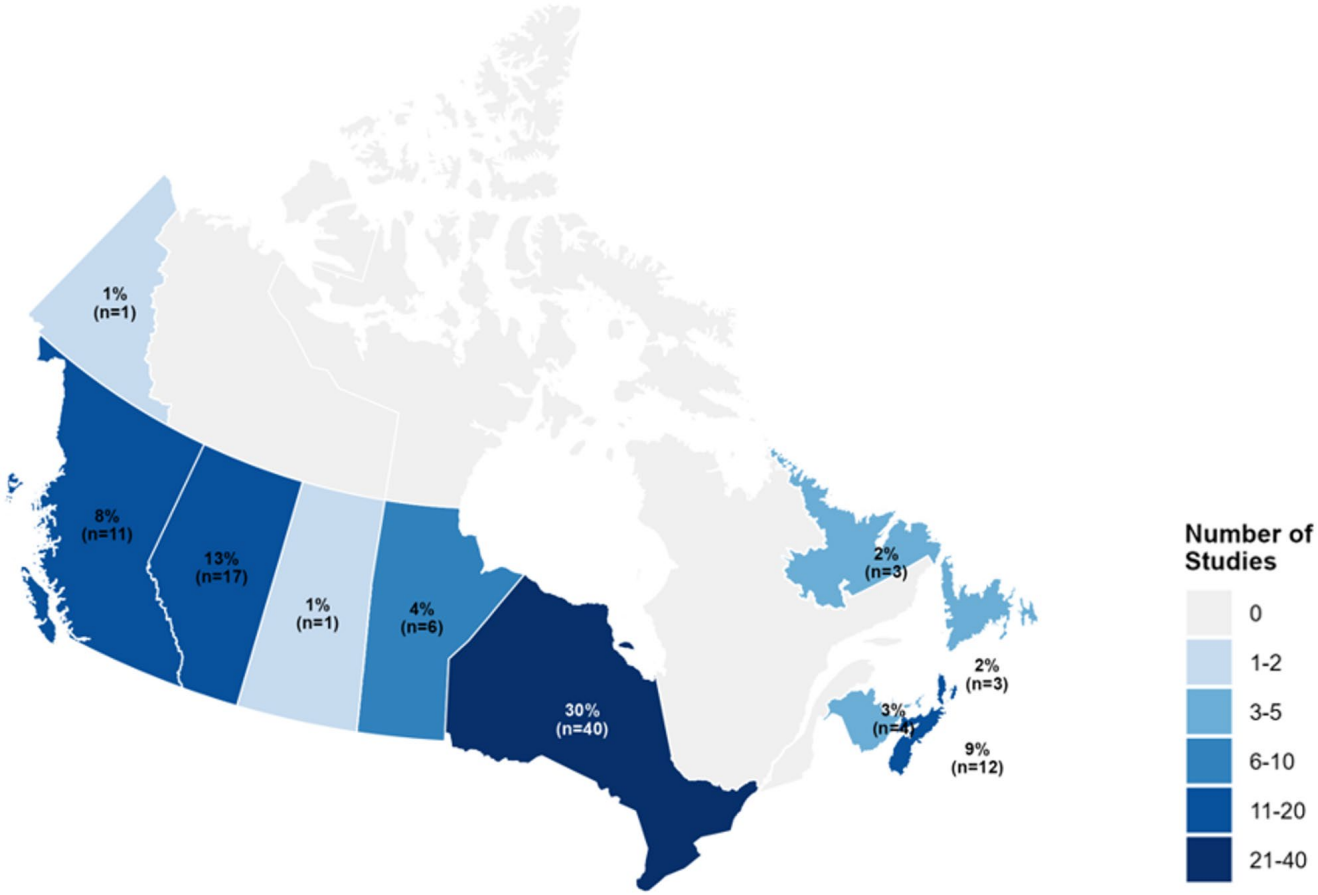
Geographic Distribution of Included Studies in Canada (*N* = 134). Darker shading represents higher study counts. An additional 38 studies examined Canada at a national level, and 21 were global in scope with a Canadian component; these are not represented on the map. Figure generated using R (v4.5.1)

**Fig. 3:**
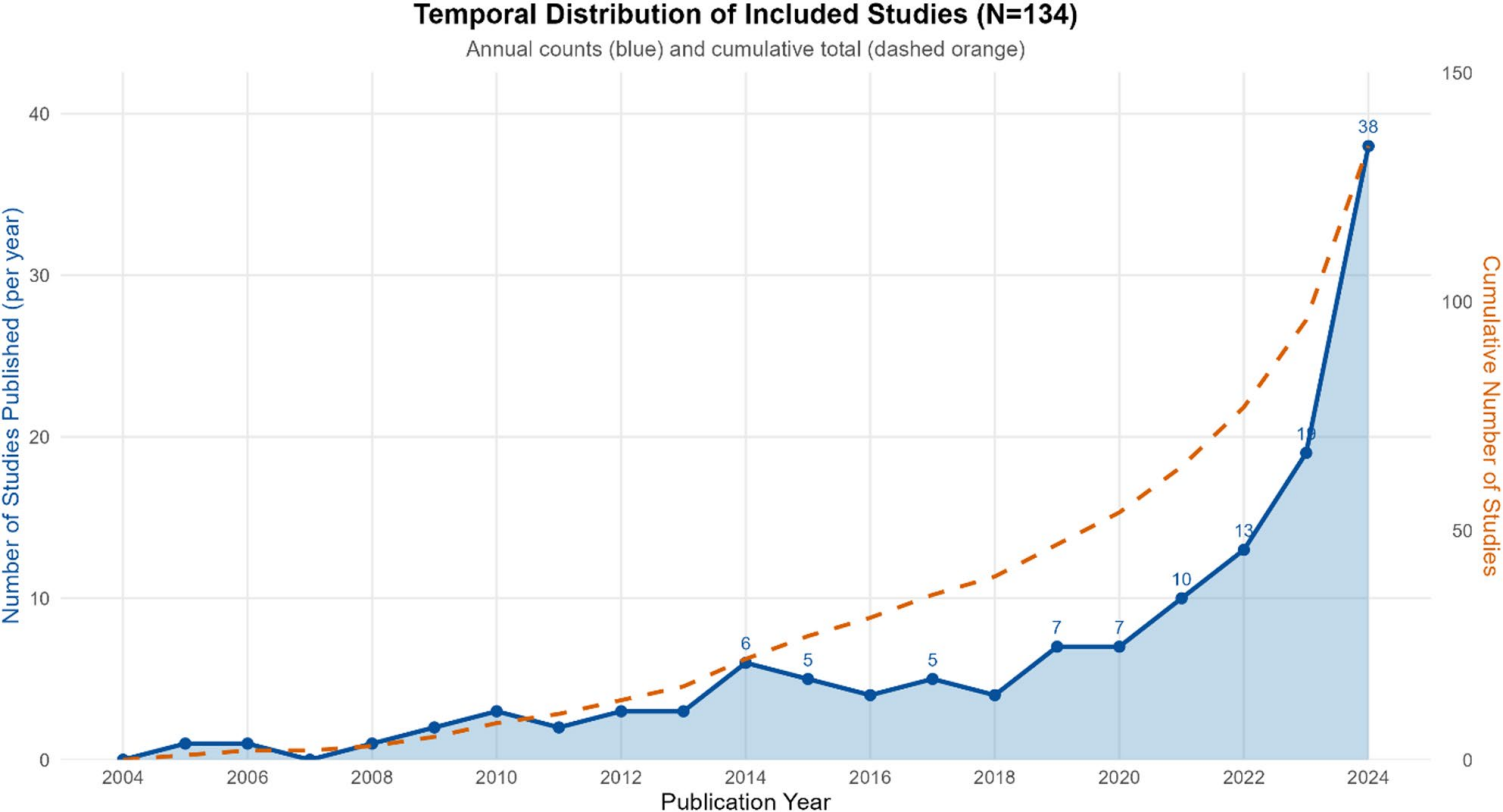
Temporal Distribution of Included Articles by Year (2004–2024). Blue line and shaded area represent annual publication counts. Dashed orange line indicates cumulative publications. Figure generated using R (v4.5.1)

Included studies revealed geographic skew that favours larger provinces (Fig. 2). Province-specific studies are geographically concentrated in Ontario (*n* = 40), Alberta (*n* = 17), Nova Scotia (*n* = 12) and British Columbia (*n* = 11), with only one study conducted in the northern territories (Fig. 2). Regarding timeframe, most articles were published between 2021 and 2024 (*n* = 115) with few articles published between 2004 and 2013 (*n* = 16) (Fig. 3).

### Institutional barriers to vaccine access

We identified several institutional factors that limit vaccine access in Canada: vaccine supply and availability, funding and coordination structures, geographic constraints, regulatory prioritization, vaccine hesitancy arising from institutional mistrust, and information availability.

### Vaccine supply and availability

Inconsistent vaccine supply availability was identified as a recurring barrier within much of the literature with multiple contributing factors. Fonseca et al. (2021) found that pharmacies experienced frequent vaccine supply shortages and inventory disruptions [29]. Houle et al. (2024) reported that restricted access to publicly funded vaccine supply contributed to missed vaccination opportunities [30]. Vaccine manufacturing and distribution challenges were also identified as contributing to limited availability especially during COVID-19 [31–34], largely attributable to Canada’s reliance on international production and its limited domestic manufacturing capacity [32].

### Funding and coordination structures

Jurisdictional funding and coordination structure(s) were additional barriers to vaccine access in Canada. Vaccines were categorized under two funding models: publicly funded vaccines provided at no cost to patients, and non-publicly funded vaccines that are excluded from provincial immunization programs [35, 36]. The absence of a nationally standardized immunization schedule has produced substantial interprovincial differences in both vaccine availability and funding allocation, driven by differences in regional health budgets, policy priorities, and jurisdictional decision-making [36, 37]. Interprovincial variations in recombinant zoster vaccine (RZV) coverage and universal seasonal influenza vaccine programs are examples of disparities that resulted from this decentralized structure (38–40). Similarly, access to the Tdap vaccine during pregnancy has also been inconsistent due to the lack of a nationally coordinated implementation strategy [38, 39].

### Geographic constraints

Geographic constraints, while infrastructural in nature, function as institutional barriers when they arise from policy decisions. Placement of vaccination services, allocation of resources, and selection of priority delivery regions are all examples of institutional factors that give rise to access barriers when combined with geographic constraints [40–42]. Houle and Eurich (2022) identified geographic disparities in pharmacist immunization access [30]. Rambout et al. (2014) reported transportation as a barrier for HPV vaccination amongst adolescents [43], and Moscou et al. (2024) reported that inadequate transportation limited childhood vaccination attendance [44]. MacDonald et al. (2022) described a remote First Nations community with one main health centre and no public transportation and low vaccination rates [45]. Kaposy and Bandrauk (2012) [46] found that incarcerated populations were inconsistently included in provincial vaccine prioritization. Moreover, the concentration of vaccination services in urban centres further constrains vaccine access. Charland et al. (2014) found that clinic proximity predicted vaccine uptake at the community level [47]. Lyeo et al. (2023) found that public transit users were more likely to experience transportation-related barriers to healthcare access [48].

### Regulatory prioritization

Regulatory prioritization mechanisms were identified as important structural factors shaping vaccine access in Canada, with the H1N1 influenza and COVID-19 pandemics serving as prominent examples. During both public health emergencies, the Public Health Agency of Canada (PHAC) and the National Advisory Committee on Immunization (NACI) issued sequential priority recommendations for vaccine allocation that target populations at elevated risk of severe disease or onward transmission [37, 46, 49–51]. Although these frameworks aimed to ensure that the most vulnerable populations were immunized first, the pace and frequency of revisions to eligibility criteria created confusion among both the public and healthcare providers [52]. Implementation was also uneven across provinces due to province-specific prioritization strategies [53, 54].

### Vaccine hesitancy

Vaccine hesitancy, defined as “the delay in acceptance or refusal of vaccination despite availability of vaccination services”, emerged as a significant behavioural barrier to vaccine access and uptake in Canada [55]. While often considered as an individual psychological phenomenon, vaccine hesitancy is analyzed as a behavioural response to institutional factors in this analysis. This framing aligns with literature recognizing that institutional mistrust is more strongly associated with vaccine hesitancy than interpersonal mistrust, and that institutional mistrust and healthcare refusal among marginalized groups reflects rational responses to institutional harm [9, 26]. Identified literature substantiates this framing through specific documentations of institutional harm and the resulting vaccine hesitancy across multiple populations.

Indigenous and Black communities have both reported mistrust in vaccines and the broader health structures due to historical and continuing racism, injustices, and unethical practices [44, 56–58]. Within Indigenous communities, vaccine mistrust has been traced to colonial violence, discriminatory practices, systemic mistreatment, and inadequate cultural safety training within healthcare systems [44, 58–60]. Tinessia et al. (2024) linked vaccine confidence among Indigenous peoples to historical colonial violence and ongoing institutional distrust [58]. Burnett et al. (2020) found that forced vaccination in residential school contexts created intergenerational trauma that persists as a barrier to trust [61]. Similarly, Malkin et al. (2024) reported that colonization-related trauma discouraged trust in government-provided care [60]. Ashfield et al. (2023) further observed that vaccine prioritization of Indigenous and older populations paradoxically eroded trust, with some perceiving prioritization as government experimentation [62]. Within Black communities, historical injustices such as the Tuskegee Syphilis Study continue to influence contemporary vaccine hesitancy [62]. Among Black communities, Ezezika et al. (2024) identified racial discrimination in healthcare as a barrier [56]. Moscou et al. (2024) found that racism in healthcare deterred access for both Black and Indigenous patients [44]. Other vulnerable populations face similar dynamics that contribute to vaccine hesitancy. Newcomers and people who use drugs reported encountering discrimination through stigmatization, criminalization, and insufficient culturally responsive care, which collectively impede vaccine access and reinforce hesitancy [31, 63, 64].

### Access to information

Access to reliable and timely information represents another barrier influencing vaccine access and uptake in Canada. While information access may appear to be an individual-level concern, it functions as an institutional barrier when shaped by governance decisions on production and dissemination of vaccine-related information. Decisions on public communication strategies, such as language, format and distribution channels, directly determine the quality and reach of vaccine information available to different populations. A public health agency that disseminates vaccine safety information exclusively through digital platforms, for instance, excludes populations without internet access or sufficient literacy to engage with such materials. Across diverse settings and populations, included studies revealed multiple dimensions of information inadequacy as obstacles to vaccine uptake. Stratoberdha et al. (2022) found that lack of vaccine information and unawareness of vaccine existence were the most frequently reported barriers to adult vaccine access [10]. Vernon-Wilson et al. (2023) found that inconsistent COVID-19 vaccine policy generated confusion and undermined public confidence [65]. Similarly, Simms et al. (2023) reported that inconsistent government messaging created access barriers for Métis communities [34]. Aylsworth et al. (2022) found that public health information required specific literacy and technology skills that excluded linguistic minorities [66]. Humble et al. (2023) attributed vaccination inequities among newcomers partly to language barriers in communications [64]. Dube et al. (2022) further found that language barriers hindered COVID-19 vaccination among BIPOC communities [67].

### Institutional factors facilitating access

We identified four key institutional facilitators to vaccine access in Canada: Scope-of-practice expansions, digital and administrative infrastructure, targeted vaccine initiatives, and inter-institutional collaboration.

### Scope of practice expansions

Efforts to increase vaccination capacity in Canada have included expanding the scope of practice to broaden the pool of qualified vaccinators beyond physicians and nurses. Pharmacists have been the primary focus of these initiatives, with nine provinces implementing legislation enabling pharmacist-administered vaccination [68]. Pharmacist-administered vaccination has been identified as a facilitator of access by offering extended hours, walk-in availability, and broader geographic coverage [65, 69, 70], with evidence demonstrating improved influenza vaccination rates in provinces that have adopted such policies [29, 71–73]. Midwives have also been authorized to administer vaccines such as Tdap at perinatal care sites [38]. However, there was again variation in the provincial regulations governing which vaccines pharmacists are authorized to administer, resulting in varied availability of the types of pharmacist-delivered vaccination across provinces [74]. Doherty and Privor-Dumm (2024) also noted that midwife involvement in vaccination, though promising, remained limited in practice [72].

During the COVID-19 pandemic, several provinces further expanded the pool of authorized vaccinators to include retired physicians, qualified international medical graduates, students, allied health professionals, and trained volunteers [72, 75, 76]. This strategy was associated with high acceptance rates among both patients and healthcare providers and contributed to a substantial expansion of vaccine access under emergency circumstances [72, 76].

### Digital and administrative infrastructure

A range of digital and administrative improvements were identified as facilitators of vaccine access in Canada. Digital platforms for appointment scheduling, automated reminders, and electronic health record-sharing enhanced accessibility, while streamlined documentation requirements and clinic workflows reduced administrative barriers [59, 77]. In Ontario and Alberta, digital reminder systems were associated with increased attendance at routine vaccination appointments and improved completion rates for multi-dose vaccine regimens [30, 78, 79].

However, the benefits of these technological advances were found to be unevenly distributed. Limited digital literacy and access to technology constrain the reach of such digital facilitation strategies, especially in marginalized communities [59, 63, 77]. The absence of centralized electronic health records also makes immunization tracking incoherent across jurisdictions [69]. Research from Ontario and Alberta further suggests that real-time access to immunization registries would enable pharmacists to identify and address gaps in vaccination coverage more effectively [33].

### Targeted vaccine initiatives

Targeted vaccination initiatives tailored to population-specific settings have demonstrated success in addressing barriers to uptake and increasing coverage among hard-to-reach populations. These initiatives typically prioritize community leadership and localized engagement strategies.

School-based vaccination programs were the most extensively implemented targeted intervention in Canada. All ten provinces operate voluntary school-based models and link vaccination to school or childcare entry requirements [80–82]. Gallant et al. (2024) reported that school-based programs addressed primary care shortages in the Maritime provinces [83]. Lind et al. (2015) found that these programs reduced demands on parental resources by eliminating the need to schedule appointments and take time off work [84]. Musto et al. (2013) found that in-school HPV vaccination delivery in Calgary achieved higher uptake than community-based public health clinic models [85]. However, Malkin et al. (2022) identified challenges in obtaining parental consent forms and insufficient capacity for school nurses to administer immunizations [60]. Lissinna et al. (2024) further noted that school-based programs were limited to school-attending children, neglecting under immunized children who are hospitalized [86].

Beyond school-based programs, mobile and pop-up vaccination clinics within alternative institutional settings have expanded access across multiple underserved populations. Tinessia et al. (2024) found that community-controlled, culturally safe vaccination clinics led by Indigenous health professionals facilitated trust and uptake in Indigenous communities [58]. Santangelo et al. (2024) reported that mobile vaccination units improved vaccine access equity by bringing services directly to underserved communities [87]. Thambinathan et al. (2024) described a hospital-shelter partnership in Toronto that improved vaccine delivery to homeless populations [88]. Similarly, Buccieri and Gaetz (2013) found that community outreach clinics in homeless shelters and drop-in centres were the most effective strategy for reaching people experiencing homelessness [49].

### Inter-institutional collaboration

Inter-institutional collaboration among healthcare agencies, community organizations, and government entities was identified as a facilitator to vaccine access and acceptability amongst diverse populations. For instance, Nickel et al. (2024) found that collaborative advocacy between First Nations leadership and the Manitoba government improved vaccine access for communities facing systemic barriers [54]. Kadio et al. (2024) identified facilitators for public health-faith-based organization collaboration, including pre-existing community relationships, co-designed strategies, and mobilizing community leaders [89]. Valaitis et al. (2020) further found that primary care and public health collaboration leveraged pre-established community relationships to deliver services to hard-to-reach populations [90]. Importantly, community-based collaborative models have demonstrated effectiveness in reaching marginalized populations. During the COVID-19 pandemic, partnerships among hospitals, community health organizations, and social services increased vaccine access for individuals experiencing homelessness [62, 88, 91], while collaborations with immigrant-serving agencies, faith-based organizations, and ethnocultural community groups reduced cultural, linguistic, and trust-related barriers for newcomer, immigrant, and refugee populations [31, 59, 92].

Indigenous-led collaborations emerged as another distinct and significant driver of vaccine access. In Alberta and Manitoba, First Nations and Métis communities partnered with provincial governments to implement culturally responsive vaccine strategies that addressed both cultural and geographic barriers [54, 58]. Similarly, government engagement with Black-led organizations, faith leaders, and healthcare providers enhanced vaccine uptake and confidence among African, Caribbean, and Black communities during the COVID-19 pandemic [93].

Though such community-led efforts have been effective in expanding vaccine access, evidence indicates such programs continue to struggle with chronic underfunding and limited support from provincial and federal governments [94, 95]. These constraints limit the scalability and sustainability of such programs.

## Discussion

We identified six barriers and four facilitators as institutional factors influencing vaccine access, covering structural, behavioural and informational dimensions across different populations. Our findings align with COVID-19 pandemic response literature emphasizing culturally tailored interventions to improve vaccine access among marginalized populations [9]. Evidence supports mobile clinics, community partnerships, and targeted mitigation of healthcare discrimination including racism, homophobia, transphobia, and ableism [9]. Pharmacists are also well-positioned to improve vaccine access and education through extended operation hours and community presence to enhance access and health literacy [96]. Our findings align with international institutional vaccine access frameworks. The WHO’s Immunization Agenda 2030 (IA2030), endorsed by the World Health Assembly in 2020, is organized around seven strategic priorities including integration of immunization within primary health care, governance coordination, and equity in access [97]. IA2030 identifies governance fragmentation, insufficient community engagement, and failure to reach “zero-dose” populations as core barriers, and emphasizes country-owned, partnership-based, and data-guided approaches to address them [97]. The OECD’s COVID-19 primary health care systems analysis similarly identifies weak coordination as a barrier to vaccination coverage, and highlights the need for expanded scope of practice and better digital health infrastructure [98]. These international parallels suggest that the institutional barriers identified in this review are not limited to the Canadian context. Our findings extend prior population and context-specific reviews to comprehensively examine both routine and pandemic immunization across a 20-year period in Canada, which captured preexisting institutional factors outside of emergency and population-specific contexts. Our inclusion of grey literature proved essential for capturing additional institutional factors that are underrepresented in peer-reviewed research, as program-level innovations such as mobile clinics, extended pharmacy scope-of-practice, and community health partnerships were documented primarily in government reports and public health agency reports. However, although grey literature credibility was assessed for source authority and relevance according to JBI guidelines, these findings should still be interpreted with attention to the potential for reporting bias, as reports may not carry the same methodological rigor as peer-reviewed literature and may emphasize program successes over limitations.

### Interactions between factors

While each institutional factor was identified as an independent determinant of vaccine access, interactions between factors also emerged. For example, inconsistent vaccine availability and funding constraints are mutually reinforcing. When provinces and territories deprioritize adoption of federally recommended vaccines due to fiscal pressures, the reduced political urgency results in supply gaps and perpetuates a cycle of underfunding and limited availability. Funding constraints also interact with geographic constraints in a mutually reinforcing manner. In remote communities, per-capita vaccine delivery costs rise sharply due to expensive cold chain logistics, but bulk purchasing agreements and funding models rarely account for these added costs [99, 100]. Vaccine hesitancy interacts with both information access and regulatory prioritization. Populations with limited access to culturally and linguistically appropriate health information are more susceptible to misinformation [101], and when lack of regulatory prioritization signal institutional uncertainty about a vaccine, that ambiguity can reinforce existing hesitancy among communities already facing informational barriers [102].

These interactions suggest that institutional barriers can compound sequentially to result in disparities in vaccine access outcomes. Formal institutional decisions function as upstream determinants, where variations in funding and distribution create structural gradients in vaccine availability that disproportionately affect marginalized communities. These formal decisions then shape the conditions under which informal institutions arise. For instance, communities that have experienced exclusion from vaccine programs develop institutional mistrust that persists even when access is later expanded [26]. This effect is further reinforced when service delivery design embeds exclusionary assumptions. Examples of such designs include digital booking platforms that presume internet access and digital literacy and centralized clinic locations that presume reliable transportation. The result is a layered process in which each institutional decision narrows access for the same populations, such that downstream barriers are not independent of, but produced by, upstream ones.

Certain facilitators also interact with multiple barriers simultaneously. Expanding pharmacist scope of practice and targeted vaccination campaigns can address both availability gaps and geographic constraints, especially for remote communities that rely on local pharmacies as their primary point-of-care. Technological advances in digital immunization databases can potentially improve cross-jurisdiction coordination to improve surveillance and continuity of care for mobile populations. Inter-institutional collaboration between public health agencies and community organizations is the most direct lever against vaccine hesitancy and information access, since trusted community partners can translate institutional messaging into culturally grounded communication that national campaigns cannot replicate at scale. The presence of these interactions suggests that interventions targeting multiple interacting barriers are more likely to produce meaningful vaccine access outcomes.

### Political economy considerations

Institutional factors cannot be examined in isolation without contextualizing the political economy of vaccine access. Politically, federal-provincial jurisdictional division is a recurring issue underlying multiple barriers. Responsibilities in operating vaccination programs are divided between federal and provincial health authorities in Canada [103]. The federal government negotiates bulk procurement and regulates market authorization through Public Health Agency of Canada and Health Canada, while provinces retain authority over funding decisions, delivery infrastructure, and scope of practice regulations [103].

This division, while reducing certain burdens from each level of government, produces significant inconsistencies identified in our findings in terms of rollout schedules and vaccine inclusion. The economic market dynamics further complicate access at the supply level, as pharmaceutical manufacturers’ pricing strategies and supply allocation decisions largely operate outside of public governance [104]. Canada’s limited domestic production capacity was an identified vulnerability grounded in this market dynamic. These political-economic dimensions suggest that corresponding attention to federal-provincial coordination and market regulations are also important considerations when addressing institutional factors.

### Theoretical considerations

While North’s institutional framework was useful in distinguishing formal and informal institutional determinants, its effectiveness is limited in examining the role of institutional actors and the power relations that create or resist institutional arrangements [6]. For example, pharmaceutical manufacturers’ pricing decisions shape vaccine supply outside of public governance, and community organizations serve as intermediaries bridging access gaps for marginalized populations. The role of such actors and the power dynamics between them are important considerations when interpreting the causes and effects of institutional barriers. Moreover, North’s distinction between formal and informal institutions offers a somewhat static categorization that does not fully capture institutional legacies. For example, vaccine mistrust amongst Indigenous communities stems from colonial violence and residential school experiences, which consist of both formal and informal institutions that cannot be effectively dichotomized into either category. Since government policies, political ideologies and legacies of systemic racism continue to shape vaccine acceptance in Canada, there is a need for complementary approaches to account for how these institutional legacies produce present-day access inequities [105]. Historical institutionalism and path dependency analysis, drawn from other schools of thought, such as public administration theories, can likely offer potential next steps for extending North’s framework to address this line of inquiry.

To partially mitigate these limitations, we supplemented North’s framework with the PROGRESS-Plus equity framework to examine where institutional factors intersect to produce access inequities. However, future research would benefit from integrating perspectives from public administration theory and institutional actor analysis to move toward understanding the organizational processes and power dynamics that sustain or transform institutional barriers to vaccine access.

### Implications for equity

We applied the PROGRESS-Plus equity framework to analyze how identified institutional barriers and facilitators inequitably distribute across populations [28, 106]. PROGRESS-Plus categorizes characteristics associated with health inequities (e.g., ethnicity, occupation, gender, education, socioeconomic status) alongside vulnerability markers such as housing instability or incarceration [28, 106]. It is well suited to supplement the equity dimension of our findings because it captures intersecting dimensions of disadvantage that aligns with the interacting nature of institutional factors identified in our review. Our findings reveal that institutional barriers cluster among populations marginalized across multiple PROGRESS-Plus categories simultaneously. Indigenous communities, for instance, are constrained by both geographic remoteness, discrimination, and systematic exclusion from vaccination program design.

Recent immigrants encounter language barriers that render English- and French-only booking platforms inaccessible, precarious employment that restricts availability during clinic hours, and unfamiliarity with provincial healthcare navigation. People experiencing homelessness contend with lack of fixed residence for appointment-based systems and limited social capital to access informal health information networks. This clustering indicates that institutional arrangements produce disproportionate barriers through cumulative disadvantage where each additional axis of marginalization exponentiates existing access barriers.

### Transferability of facilitators beyond pandemic contexts

The predominance of COVID-19–era literature in our synthesis raises questions about facilitator sustainability beyond emergency contexts. We distinguish short- and long-term interventions among identified facilitators based on their structural characteristics and post-pandemic trajectory. Temporary pandemic adaptations include the creation of mass vaccination sites, emergency regulatory authorizations such as the temporary expansion of scope of practice to permit lay student vaccinators, retired physicians, and trained volunteers to administer vaccines, and temporary inter-institutional coordination measures [7, 76]. These interventions are designed to respond to unprecedented acute circumstances under compressed timelines that differ from routine immunization. Mass vaccination sites have largely been decommissioned, and the political will sustaining intensive federal-provincial coordination has diminished as pandemic urgency receded [107, 108]. These adaptations offer limited direct transferability to routine programs, though they generated operational knowledge about high-volume delivery that may inform future emergency preparedness.

Interventions with sustained long-term potential include expanded vaccinator scope of practice, community health and pharmacy-based immunization infrastructure, and digital infrastructure improvements. Legislative changes enabling pharmacist vaccination have remained in effect post-pandemic across most provinces, and pharmacy networks have become new vaccine access points through integration of immunization into routine service offerings.

Digital appointment systems, while initially deployed for COVID-19, have been adapted for influenza and other routine vaccines in several provinces [109]. Moreover, community-based interventions such as mobile vaccination units and community organization partnerships demonstrated effectiveness in reaching marginalized populations. Their sustainability, however, will depend on long-term funding availability and organizational commitment beyond emergency allocations. Many mobile programs have been scaled back or discontinued as targeted funding expired following the COVID-19 pandemic.

### Limitations

Scoping reviews do not typically assess the quality of risk of bias of the included documents, meaning findings could include documents with methodological limitations or limited generalizability. Methodological limitations beyond inherent scoping review design limitations include language restriction, varied timeframe, COVID-19 concentration, geographic skew and specificity. The English-language restriction resulted in potential underrepresentation of Quebec’s Francophone literature, Indigenous-language documentation, and community resources produced in immigrant languages. The differential timeframes applied to peer-reviewed (2004–2024) and grey literature (2014–2024) may have excluded relevant older policy documents, though this approach prioritized currency in rapidly evolving governmental guidance. Additionally, though we aimed to examine literature beyond that specific to the COVID-19 pandemic, most identified documents were published on this issue. This may introduce publication bias as emergency conditions differ from routine immunization. Moreover, the geographic concentration of included literature in Ontario and Alberta limits the transferability of findings to other jurisdictions. These two provinces benefit from larger research infrastructure and funding capacity, which produces an evidence gap where regions with the fewest resources and most distinct vaccine access challenges (e.g., northern territories) are the least represented in literature. Consequently, the institutional barriers and facilitators identified in this review may not adequately reflect the experiences of underrepresented regions. Similarly, while the review’s targeted grey literature searches focused on populations served by federal institutional pathways (Indigenous communities, veterans, and refugees), other populations facing institutional barriers to vaccine access may be underrepresented in our grey literature findings.

This reveals the need for targeted research investment in generating territorial-specific health data to inform tailored recommendations. Lastly, our aggregated analysis across vaccine types and platforms, while comprehensive, represents an additional methodological trade-off, as it cannot fully account for how institutional determinants operate differently across the life course. Our analysis, however, situated certain life course-specific mechanisms within the broader institutional typology. For instance, school-based delivery platform is most relevant as a facilitator to childhood routine immunizations, and vaccine hesitancy manifests as a barrier within the parental consent component. Adult and senior vaccines are subject to funding barriers, where gaps in provincial coverage shift financial responsibility to individuals and produce income-stratified access patterns. These examples show that while the institutional determinants identified in our framework operate across vaccine types, the specific mechanisms through which they shape access do vary by life course stage. Our findings can be expanded through application to specific vaccine types or age cohorts to yield more granular life-course insights. Future research should prioritize territorial jurisdictions, examine long-term sustainability of pandemic-implemented facilitators, and employ comparative effectiveness designs evaluating intervention impact across population subgroups and delivery settings.

Our review identified theoretical gaps that warrant attention in future analyses of institutional factors influencing vaccine access. To address limitations posed by North’s institutional framework, future research should examine further the institutional actors involved and the power dynamics that produce or resist the factors identified in this review. How colonial structures and path-dependent policy trajectories continue to shape contemporary vaccine access is also a potential direction for further research. Finally, comparing between Canadian provinces and territories would help explain why similar formal institutions produce different access outcomes, which would better account for the influence of informal institutions at the local level.

### Preliminary implications of findings

The institutional barriers identified in this review point to three priority intervention domains. First, enhanced inter-institutional and cross-jurisdictional collaboration would harmonize vaccine policy and reduce provincial/territorial disparities. The current decentralized model creates inequities for marginalized communities that undermines national immunization objectives. Second, national equity-centered standards supported by sustainable funding for community-based and primary care delivery would address structural barriers. Third, healthcare system strengthening requires expanded access points, modernized data infrastructure for real-time tracking, and workforce training to support equitable and culturally safe delivery.

## Conclusions

This scoping review provides a high-level overview of the institutional factors influencing vaccine access and uptake in Canada [26–92]. Thematic analysis of academic and grey literature identified six institutional barriers, and four facilitators as described in the included literature. These findings support three policy priorities: a standardized pan-Canadian immunization framework to ensure consistent coverage across provinces, a centralized national immunization registry to enable real-time surveillance and reduce data fragmentation and sustained federal funding mechanisms for community-based delivery models that have demonstrated effectiveness in reaching populations facing intersecting access barriers.

Examining institutional determinants is necessary to advance vaccine equity, since institutional structures can either reduce or deepen existing inequities. This review identifies concrete avenues for strengthening vaccine programs in Canada to better reach marginalized and underserved populations. Inter-institutional collaboration among healthcare organizations, government entities, and community partners requires fortification and expansion.

Collaborative frameworks demonstrated effectiveness in enhancing vaccine access for populations facing structural barriers. Further, there is a clear need for national-level standards or frameworks to create coherence across provinces and territories. This can be combined with sustainable funding models and health systems strengthening initiatives to improve vaccine access at the structural level. The COVID-19 pandemic exposed important gaps in Canada’s existing vaccine infrastructure. Systematic understanding of institutional factors enables development of resilient, responsive systems capable of addressing routine immunization requirements and emerging public health threats.

## Appendix A

**Table:** Academic and grey literature search strategy

Database	Query String
Scopus	TITLE-ABS ( ( vaccin* ) AND ( access OR availability ) ) AND PUBYEAR > 2004 AND PUBYEAR < 2024 AND Canada AND ( LIMIT-TO ( DOCTYPE,“ar” ) OR LIMIT-TO ( DOCTYPE,“re” ) ) AND ( LIMIT-TO ( LANGUAGE,“English” ) ) AND ( EXCLUDE ( EXACTKEYWORD,“Animal” ) )
Ovid Embase	((“vaccin*” or “immunotherap*”) and (“access” or “availability”) and Canada) limit 1 to (english language and humans and yr="2004–2024”)
Ovid Medline/Google Advanced Search (grey literature)	1. exp Vaccines/ 2. exp Immunotherapy/ 3.vaccin*.tw, kf. 4. immunotherap*.tw, kf. 5.access*.tw, kf. 6.availabilit*.tw, kf. 7.(or/1–4) and (or/5–6) 8. (Animals/ or Models, Animal/ or Disease Models, Animal/) not Humans/ 9. ((animal or animals or canine* or dog or dogs or feline or hamster* or lamb or lambs or mice or monkey or monkeys or mouse ormurine or pig or pigs or piglet* or porcine or primate* or rabbit* or rats or rat orrodent* or sheep* or veterinar*) not (human* or patient*)).ti, kf, jw. 10. 8 or 9 11. 7 not 10 12. exp Canada/ 13. (canadian* or canada* or british columbia* or alberta* or saskatchewan* or manitoba* or ontario* or quebec* or new brunswick* or prince edward island* or nova scotia* or labrador* or newfoundland* or nunavut* or northwestterritor* or yukon* or toronto* or montreal* or vancouver* or ottawa* or calgary* or edmonton* or winnipeg* or first nation* or metis).ti, ab, hw, kf. 14. 12 or 13 15. 11 and 14

## Appendix B

**Table:** Targeted website searches

Targeted Searches of Government Websites		
Scope	Organization	
National	Health Canada	https://www.canada.ca/en/health-canada.html
	Canadian Drug Agency	https://www.cda-amc.ca/
	National Advisory Committee on Immunization (NACI)	https://www.canada.ca/en/public-health/services/immunization/national-advisory-committee-on-immunization-naci.html
	Public Health Agency of Canada (CIG)	https://www.canada.ca/en/public-health/services/immunization/national-advisory-committee-on-immunization-naci.html
Provincial	Health Quality Ontario (HQO)	https://www.hqontario.ca/
	Ontario Ministry of Health (MOH)	https://www.ontario.ca/page/ministry-health
	Quebec Ministry of Health	https://www.msss.gouv.qc.ca/en/
	Government of Quebec	https://www.quebec.ca/en
	British Columbia Ministry of Health	https://www2.gov.bc.ca/gov/content/governments/organizational-structure/ministries-organizations/ministries/health
	Alberta Ministry of Health	https://www.alberta.ca/health
	Alberta Health Services	https://www.albertahealthservices.ca/
	Manitoba Ministry of Health	https://www.gov.mb.ca/health/
	Shared Health Manitoba	https://sharedhealthmb.ca/
	Government of Newfoundland & Labrador	https://www.gov.nl.ca/
	Newfoundland Health Services	https://nlhealthservices.ca/
	Health PEI	https://www.princeedwardisland.ca/en/topic/health-pei
	New Brunswick Department of Health	https://www2.gnb.ca/content/gnb/en/departments/health.html
	Government of Nova Scotia	https://novascotia.ca/dhw/
	Nova Scotia Health	https://www.nshealth.ca/
	Ministry of Health, Government of Saskatchewan	https://www.saskatchewan.ca/government/government-structure/ministries/health
Territorial	Government of Yukon	https://yukon.ca/en/department-health-social-services
	Government of Nunavut	https://www.gov.nu.ca/en/health
	Government of Northwest Territories	https://www.hss.gov.nt.ca/en

Targeted Hand Searches		
Scope	Organization	Website﻿
International Organizations	Pan American Health Organization (PAHO)	https://www.paho.org/en
	United Nations (UN)	https://www.un.org/en/
Indigenous Organizations	Indigenous Services Canada – Vaccinations for First Nations and Inuit	https://www.sac-isc.gc.ca/eng/1626810177053/1626810219482#sec1
Veterans and Military	Veterans Affairs Canada – Prescription Drug Program (POC 10)	https://www.veterans.gc.ca/en/financial-programs-and-services/medical-costs/search-prescription-drug-program-poc-10
Refugees	Interim Federal Health Program – Summary of Coverage	https://www.canada.ca/en/immigration-refugees-citizenship/services/refugees/help-within-canada/health-care/interim-federal-health-program/coverage-summary.html
Correctional Services	Government of Canada: Correctional Services Canada	https://www.canada.ca/en/immigration-refugees-citizenship/services/refugees/help-within-canada/health-care/interim-federal-health-program/coverage-summary.html
Additional Sources	Provincial and Territorial Immunization Information	https://www.canada.ca/en/public-health/services/provincial-territorial-immunization-information.html
	Vaccine Supply – Government of Canada	https://www.canada.ca/en/public-health/services/vaccine-supply.html

## Supplementary Information

Supplementary Material 1

## Data Availability

All relevant data is available in the publications included in the reference list of this manuscript. A summary table of all included studies is attached as Appendix C.
