# Supplementary material for: Institutional factors influencing vaccine access in Canada: a scoping review
Source: BMC Public Health. 2026 Apr 29;26:2030. doi: 10.1186/s12889-026-27593-w (PMC13330069; doi:10.1186/s12889-026-27593-w)
Supplement: Supplementary file 1 — Supplementary Material 1 [file 12889_2026_27593_MOESM1_ESM.docx]

**Appendix C. Summary Table of Included Study Characteristics**

| Author(s), Year | Title | Vaccine Type | Population and Sample Size | Location | Institutional Factors (North) | Key Findings |
| --- | --- | --- | --- | --- | --- | --- |
| Aghajafari et al., 2024 | An exploration of COVID-19 vaccination models for newcomer refugees and immigrants in Calgary, Canada. | COVID-19 | Government-assisted refugees (n=39), privately sponsored refugees (n=6), sponsors (n=3), stakeholders (n=13) | Alberta | Formal: Health System Governance; Formal: Service Delivery Design; Informal: Inter-organizational Coordination | Vaccine undersupply was a structural barrier. Non-governmental partnerships facilitated outreach to newcomer communities. Tailored delivery through community-based and culturally accessible locations improved vaccination uptake. |
| Aghajafari et al., 2024 | COVID-19 Vaccinations, Trust, and Vaccination Decisions within the Refugee Community of Calgary, Canada. | COVID-19 | Afghan refugees and refugee sponsors (n=61) | Alberta | Formal: Health System Governance; Formal: Service Delivery Design; Informal: Cultural Constraints; Informal: Inter-organizational Coordination | Determinants of undervaccination included structural, legal, and informational factors. Trusted intermediaries delivering timely information in environments responsive to refugee needs facilitated vaccination. Racialized immigrants faced historical mistrust and limited vaccine accessibility. |
| Alghalyini et al., 2024 | Vaccine hesitancy among Syrian refugee parents in Canada: A multifaceted challenge in public health. | General | Syrian refugee parents (n=540) | Canada | Informal: Professional Norms (Nurses/Physicians) | Lack of a family doctor was significantly associated with vaccine hesitancy among Syrian refugees, underscoring the role of continuous primary care in building trust and promoting vaccine acceptance. |
| Ali et al., 2023 | Identifying barriers and facilitators to COVID-19 vaccination uptake among People Who Use Drugs in Canada: a National Qualitative Study. | COVID-19 | People who use drugs (n=114) | Canada | Formal: Administrative Infrastructure; Formal: Geographic and Logistical Access; Formal: Service Delivery Design; Informal: Cultural Constraints | People who use drugs faced structural marginalization limiting vaccine access. Harm reduction facilities were the most accessible vaccination settings for this population. Transportation costs and online booking requirements posed additional barriers. |
| Alsabbagh et al., 2018 | Pharmacists as immunizers, their pharmacies and immunization services: A survey of Ontario community pharmacists. | Influenza | Pharmacists (n=4,307) | Ontario | Informal: Professional Norms (Pharmacists) | Community pharmacy walk-in and appointment-based influenza vaccination during extended operating hours was identified as a key accessibility advantage over traditional provider settings. |
| Alsabbagh et al., 2019 | Pharmacy patron perspectives of community pharmacist administered influenza vaccinations. | Influenza | Pharmacy patrons aged 18+ (n=541) | Ontario | Informal: Information Access and Trust; Informal: Professional Norms (Pharmacists) | Lack of public awareness of pharmacist-administered immunization and perceived low necessity for vaccination persisted one year after service introduction, even among pharmacy patrons. |
| Ashfield et al., 2023 | Community organization perspectives on COVID-19 vaccine hesitancy and how they increased COVID-19 vaccine confidence: a Canadian Immunization Research Network, social sciences and humanities network study. | COVID-19 | Community-based organization representatives (n=41) | Canada | Formal: Regulatory Frameworks; Informal: Cultural Constraints; Informal: Inter-organizational Coordination | Pre-existing relationships with community health workers facilitated trust and vaccine uptake. Vaccine prioritization of Indigenous and older populations paradoxically eroded trust, with some perceiving prioritization as government experimentation. |
| Atkinson et al., 2016 | Can mobile technologies improve on-time vaccination? A study piloting maternal use of ImmunizeCA, a Pan-Canadian immunization app. | General | Childbearing women (n=55) | Canada | Formal: Administrative Infrastructure | A mobile immunization tracking app increased self-reported on-time pediatric vaccination likelihood for one-third of participants, with high usability ratings. |
| Aylsworth et al., 2022 | A qualitative investigation of facilitators and barriers to accessing COVID-19 vaccines among Racialized and Indigenous Peoples in Canada. | COVID-19 | Racialized (n=17) and Indigenous (n=10) individuals (n=27) | Canada | Formal: Service Delivery Design; Informal: Information Access and Trust | People with disabilities lacked appropriate accommodations at vaccination centres. Public health information required specific language, literacy, and technology skills that excluded linguistic minorities. |
| Bandara et al., 2022 | An equity-based assessment of immunization-related responses in urban Alberta during the 2014 measles outbreak: a comparative analysis between Calgary and Edmonton. | Measles |  | Alberta | Formal: Service Delivery Design | Calgary's deployment of mass immunization clinics in 2014 contributed to higher gross immunization rates compared to areas without supplementary clinics. |
| Bhatia et al., 2017 | Provincial Comparison of Pharmacist Prescribing in Canada Using Alberta's Model as the Reference Point. | General | Pharmacists (n=13) | Canada | Informal: Professional Norms (Pharmacists) | All provinces enabling pharmacist injection required formal training certification. Most allowed pharmacists to administer vaccines beyond influenza, though the scope varied considerably by jurisdiction. |
| Bhatti et al., 2022 | Using Trusted Relationships and Community-Led Approaches to Promote COVID-19 Vaccine Confidence and Uptake across Ontario. | COVID-19 | Long-term care and retirement home centres (n=45) | Ontario | Formal: Administrative Infrastructure; Formal: Service Delivery Design; Informal: Cultural Constraints | Live interpretation services improved vaccine access for linguistically diverse communities. Barriers included inadequate infrastructure for pop-up clinics, staff workload capacity, and clients' low digital literacy. |
| Brown et al., 2024 | Maternal Disability and Early Child Preventive Care | DTaP-IPV-Hib, pneumococcal conjugate, meningococcal conjugate, MMR, and varicella vaccines. | Children born 2012-2019; mothers with and without disabilities (n=831,223) | Ontario | Formal: Administrative Infrastructure; Formal: Geographic and Logistical Access; Formal: Scope-of-Practice Regulation; Informal: Cultural Constraints; Informal: Information Access and Trust; Informal: Inter-organizational Coordination | Mothers with intellectual and developmental disabilities faced barriers including negative provider attitudes, fear of judgment, transportation difficulties, and inaccessible health education. Provider training and inter-service collaboration were recommended. |
| Buccieri & Gaetz, 2013 | Ethical vaccine distribution planning for pandemic influenza: Prioritizing homeless and hard-to-reach populations | Influenza | People experiencing homelessness (n=49) | Ontario | Formal: Regulatory Frameworks; Formal: Service Delivery Design; Informal: Cultural Constraints; Informal: Information Access and Trust; Informal: Inter-organizational Coordination; Informal: Professional Norms (Nurses/Physicians) | Community outreach vaccination clinics in homeless shelters and drop-in centres were the most effective strategy for reaching homeless populations. Access to a regular doctor significantly predicted vaccination uptake. Trust-building through existing community services was essential. |
| Buchan et al., 2017 | Impact of pharmacist administration of influenza vaccines on uptake in Canada. | Influenza | Respondents aged 12+ (n=481,526) | Canada | Informal: Professional Norms (Pharmacists) | Provinces with policies allowing pharmacist-administered publicly funded influenza vaccines had higher vaccination coverage. Pharmacy-based immunization showed early positive population-level effects. |
| Burnett et al., 2020 | Indigenous Peoples, settler colonialism, and access to health care in rural and northern Ontario. | Influenza | Healthcare providers, community members, and Indigenous Elders (n=not specified) | Ontario | Informal: Cultural Constraints | Indigenous communities experienced schools as sites of conflict regarding vaccination, rooted in intergenerational trauma. Negative childhood experiences with forced vaccination in residential school contexts persisted as barriers to trust. |
| Cao et al., 2023 | Lived Experiences of the COVID-19 Pandemic Among the Vietnamese Population in the Region of Peel | COVID-19 | Community members (n=14) | Ontario | Formal: Administrative Infrastructure; Formal: Geographic and Logistical Access; Formal: Regulatory Frameworks | Prioritized groups (frontline workers, seniors) reported easier vaccination experiences. Younger, non-prioritized participants faced significant difficulties with online booking systems and geographic catchment restrictions. |
| Castillo et al., 2021 | Vaccination in pregnancy: Challenges and evidence-based solutions | Influenza |  | Global with Canadian Relevance | Formal: Administrative Infrastructure; Formal: Geographic and Logistical Access; Formal: Regulatory Frameworks; Formal: Service Delivery Design; Informal: Community-Based Delivery Norms; Informal: Cultural Constraints | Systematic exclusion of pregnant individuals from vaccine research delayed safety data. Point-of-care vaccination coupled with provider recommendation was more effective than reminders alone. Midwife-administered Tdap at perinatal care sites addressed opportunity and motivation barriers. |
| Chadi et al., 2024 | Key stakeholder perspectives on delivery of vaccination services in Quebec community pharmacies. | General | Vaccination stakeholder groups (n=14) | Quebec | Informal: Professional Norms (Pharmacists) | Pharmacy-based vaccination tools were considered adequate; however, efficiency of dispensing activities needed improvement to free time for vaccination services. Pharmacies were valued as confidential, professional environments for immunization. |
| Chambers et al., 2022 | Increases in human papillomavirus vaccine coverage over 12 months among a community-recruited cohort of gay, bisexual, and other men who have sex with men in Canada. | HPV | Gay, bisexual, and other men who have sex with men (n=1,224) | Québec, Ontario, and British Columbia | Formal: Funding Mechanisms | Age-ineligible individuals faced out-of-pocket costs up to $550 CAD for HPV vaccination. Targeted publicly funded programs for gay, bisexual, and other men who have sex with men were introduced in most provinces. |
| Charland et al., 2014 | Clinic accessibility and clinic-level predictors of the geographic variation in 2009 pandemic influenza vaccine coverage in Montreal, Canada. | Influenza | Montreal residents; population-level vaccination records | Quebec | Formal: Geographic and Logistical Access; Informal: Professional Norms (Nurses/Physicians) | Driving time to clinics was a significant factor in vaccination decisions, with 90% of trips under 15 minutes. Clinic proximity and capacity predicted vaccine uptake at the community level. |
| Chen et al., 2024 | Adult influenza vaccination coverage before, during and after the COVID-19 pandemic in Canada. | Influenza | Adults (n=3,026-5,364) | Canada | Informal: Professional Norms (Pharmacists) | Expanded pharmacy access and mass vaccination clinics established for COVID-19 facilitated broader influenza vaccine uptake, linked to pharmacists' growing authorization to administer vaccines in community settings. |
| Colmegna et al., 2021 | Barriers and facilitators to influenza and pneumococcal vaccine hesitancy in rheumatoid arthritis: A qualitative study | Influenza | Rheumatoid arthritis patients (n=28) and healthcare providers (n=26) | Quebec | Formal: Funding Mechanisms; Formal: Geographic and Logistical Access; Formal: Regulatory Frameworks; Formal: Service Delivery Design; Informal: Cultural Constraints; Informal: Information Access and Trust; Informal: Inter-organizational Coordination; Informal: Professional Norms (Nurses/Physicians) | Cost, travel distance, social media misinformation, and language barriers limited vaccine uptake among rheumatoid arthritis patients. Point-of-care vaccination at rheumatology appointments and multi-site availability were proposed facilitators. |
| Doroshenko et al., 2012 | Challenges to immunization: the experiences of homeless youth. | General | Homeless youth (n=29) | Nova Scotia | Informal: Information Access and Trust; Informal: Inter-organizational Coordination | Trusted community organizations (e.g., youth shelters) were identified as preferred settings for vaccinating homeless youth. Immunization was a low priority for this population relative to food, shelter, and employment needs. |
| Driedger et al., 2024 | "There's a little bit of mistrust": Red River Metis experiences of the H1N1 and COVID-19 pandemics. | COVID-19 | Métis adults (n=222) | Manitoba | Formal: Regulatory Frameworks | NACI recommended COVID-19 vaccine prioritization for Indigenous populations; however, Manitoba was the only province to initially include all Indigenous groups. Métis communities experienced inconsistent access despite federal prioritization. |
| Dube et al., 2015 | Promoting vaccination: implementation of targeted interventions to enhance access to vaccination services in Quebec (Canada). | General | Not specified | Québec | Formal: Program Delivery Structures; Informal: Professional Norms (Nurses/Physicians) | Recall interventions for unvaccinated children and phone reminders for unreturned school consent forms were used in most community clinics. Scheduling procedures for two-month vaccination appointments improved modestly over time. |
| Dube et al., 2022 | "I don't think there's a point for me to discuss it with my patients": exploring health care providers' views and behaviours regarding COVID-19 vaccination | COVID-19 | Vaccination stakeholders (n=14) | Canada | Formal: Administrative Infrastructure; Formal: Geographic and Logistical Access; Formal: Service Delivery Design; Informal: Cultural Constraints; Informal: Information Access and Trust; Informal: Professional Norms (Nurses/Physicians) | Language barriers and lack of locally tailored strategies hindered COVID-19 vaccination among BIPOC communities. Extended clinic hours, mobile units, home visits, and multilingual services were recommended. Trusted community nurses facilitated uptake. |
| Ecarnot et al., 2020 | Strategies to Improve Vaccine Uptake throughout Adulthood | General |  | Global | Formal: Administrative Infrastructure; Formal: Service Delivery Design; Informal: Inter-organizational Coordination; Informal: Professional Norms (Pharmacists) | Increasing provider diversity, including pharmacies, expanded vaccination access. Workplace influenza vaccination tripled uptake odds. Mobile app incentives and geolocation features showed promise for facilitating pharmacy-based vaccination. |
| Edwards et al., 2015 | Pharmacists as immunizers: a survey of community pharmacists' willingness to administer adult immunizations. | General | Pharmacists (n=495) | Canada | Informal: Professional Norms (Pharmacists) | Most Canadian pharmacists supported expanding their scope to include immunizations, though many felt undergraduate education was inadequate and formal certification should be required. |
| Egilman et al., 2021 | Optimizing the data available via Health Canada's clinical information portal | N/A | N/A | Canada | Informal: Information Access and Trust | Health Canada's public release of clinical data for regulatory submissions had low utilization despite potential to strengthen evidence-based regulatory and clinical decision-making. |
| Eren Vural et al., 2023 | Biopharmaceutical Financialization and Public Funding of Medical Countermeasures (MCMs) in Canada During the COVID-19 Pandemic. | COVID-19 | Not specified | Canada | Formal: Funding Mechanisms; Formal: Health System Governance | Canada's COVID-19 vaccine rollout was delayed by a lack of domestic mRNA manufacturing capacity. The federal government invested $415 million in a private vaccine manufacturing facility. |
| Etowa et al., 2024 | Understanding Low Vaccine Uptake in the Context of Public Health in High-Income Countries: A Scoping Review | General |  | Global | Formal: Geographic and Logistical Access; Informal: Cultural Constraints; Informal: Information Access and Trust; Informal: Inter-organizational Coordination; Informal: Professional Norms (Pharmacists) | Structural racism, lack of race-based data collection, and systemic inequities in vaccine distribution affected ACB communities. Pharmacists and racial concordance in healthcare facilitated trust. Community-led collaboration and engagement were essential. |
| Ezezika et al., 2024 | Resilience throughout COVID-19: Unmasking the realities of COVID-19 and vaccination facilitators, barriers, and attitudes among Black Canadians. | COVID-19 | Black Canadians and individuals of African descent (n=130) | Canada | Formal: Service Delivery Design; Informal: Cultural Constraints | Racial discrimination in healthcare and inaccessible vaccine locations were identified as barriers in Black communities, suggesting systemic interventions beyond vaccine supply are needed. |
| Fahim et al., 2024 | Challenges facing Canadian Long-Term Care Homes and Retirement Homes during the COVID-19 pandemic | COVID-19 | Long-term care and retirement home leadership (n=91 across 47 homes) | Ontario | Informal: Cultural Constraints; Informal: Information Access and Trust | COVID-19 vaccine challenges in long-term care included staff mistrust, booster fatigue, and perceived mandate infringements. Dedicated vaccine champions, educational resources, and public health unit support were used as mitigation strategies. |
| Falkenbach et al., 2024 | Denmark, the United States and Canada: Before, during and post vaccination rollout | COVID-19 |  | Global | Formal: Administrative Infrastructure; Formal: Funding Mechanisms; Formal: Regulatory Frameworks; Informal: Cultural Constraints; Informal: Information Access and Trust | Canada's COVID-19 vaccine rollout prioritized high-risk groups with no-cost access. High initial public trust in health officials supported uptake, but booster acceptance declined as concern about the virus waned. |
| Fonseca et al., 2021 | Distributing Publicly-Funded Influenza Vaccine-Community Pharmacies' Perspectives on Acquiring Vaccines from Public Health and from Private Distributors in Ontario, Canada. | Influenza | Pharmacists (n=not specified; response rates 45.2% and 31.4%) | Ontario | Formal: Health System Governance; Informal: Professional Norms (Pharmacists) | Pharmacies experienced vaccine supply shortages and inventory disruptions. Public-private partnerships integrating pharmacists as influenza immunizers improved vaccination rates, with pharmacies becoming the primary influenza vaccination site in several provinces. |
| Foong et al., 2017 | Ready or not? Pharmacist perceptions of a changing injection scope of practice before it happens. | General | Pharmacists, technicians, public, and organizations (n=308) | Canada | Informal: Professional Norms (Pharmacists) | Pharmacists recognized relative advantages of expanded immunization scope but expressed concerns about employer relationships, workload implications, and liability for new vaccine administration. |
| Fullerton et al., 2023 | Barriers experienced by families new to Alberta, Canada when accessing routine-childhood vaccinations. | Routine childhood vaccines | Newcomers (n=47) | Alberta | Formal: Administrative Infrastructure; Formal: Geographic and Logistical Access; Informal: Information Access and Trust; Informal: Professional Norms (Nurses/Physicians) | Newcomer families lacked knowledge of Canadian vaccination schedules and found the public health system difficult to navigate. Limited appointment availability, restricted clinic hours, and lack of a primary care provider were key access barriers. |
| Gallant et al., 2024 | Stakeholders' experiences with school-based immunization programs during the COVID-19 pandemic in the Canadian Maritimes: A qualitative study. | Routine childhood vaccines | Decision-makers, healthcare providers, teachers, parents, and adolescents (n=35) | Nova Scotia, Prince Edward Island and New Brunswick | Formal: Program Delivery Structures; Informal: Inter-organizational Coordination | School-based immunization programs addressed primary care shortages in the Maritimes. Success of these programs depended on individual relationships with school staff, which required years of investment to develop. |
| Garg et al., 2021 | COVID-19 Vaccine Hesitancy in the LGBTQ+ Population: A systematic review | COVID-19 |  | Global with Canadian Relevance | Formal: Service Delivery Design; Informal: Cultural Constraints; Informal: Information Access and Trust; Informal: Inter-organizational Coordination | Fear of discrimination, negative healthcare experiences, and distrust of authorities were barriers for LGBTQ+ populations. Community-based vaccination sites and leveraging LGBTQ+ trusted sources for accurate information were recommended. |
| George et al., 2024 | Attitudes, barriers, and facilitators to adherent completion of the recombinant zoster vaccine regimen in Canada: Qualitative interviews with healthcare providers and patients. | Recombinant zoster vaccine (RZV) | Healthcare providers and patients (n=12) | Canada | Formal: Administrative Infrastructure; Formal: Funding Mechanisms; Formal: Regulatory Frameworks | Public funding for recombinant zoster vaccine was limited to select provinces and demographic groups. Lack of follow-up appointment systems and absence of patient reminders hindered vaccine series completion. |
| Gilbert et al., 2023 | Uptake of Mpox vaccination among transgender people and gay, bisexual and other men who have sex with men among sexually-transmitted infection clinic clients in Vancouver, British Columbia. | Mpox | Transgender and gender-diverse/bisexual men (n=331) | British Columbia | Informal: Cultural Constraints | Low-barrier Mpox vaccination clinics in British Columbia addressed privacy concerns and societal stigma among sexual and gender minorities by minimizing identity disclosure requirements. |
| Grewal et al., 2021 | Human papillomavirus (HPV) vaccine uptake among a community-recruited sample of gay, bisexual, and other men who have sex with men in the three largest cities in Canada from 2017 to 2019. | HPV | Gay, bisexual, and other men who have sex with men (n=not specified) | British Columbia (BC); Ontario; and Quebec" | Formal: Administrative Infrastructure; Formal: Funding Mechanisms | Publicly funded HPV vaccination for GBM required disclosure of same-sex activity, creating a barrier. Men with private insurance were twice as likely to initiate vaccination. Bundling HPV vaccination with sexual health services was proposed to improve uptake. |
| Guttmann et al., 2006 | Volume matters: physician practice characteristics and immunization coverage among young children insured through a universal health plan. | General | Infants born in Ontario hospitals (n=101,570) | Ontario | Formal: Service Delivery Design | Higher primary care visit volumes for young children were associated with higher immunization rates, suggesting that continuity and frequency of primary care contact facilitated vaccination. |
| Haas et al., 2009 | Drugs, sex, money and power: An HPV vaccine case study | HPV |  | Global with Canadian Relevance | Formal: Funding Mechanisms; Formal: Program Delivery Structures; Informal: Cultural Constraints; Informal: Inter-organizational Coordination | All Canadian provinces adopted voluntary school-based HPV programs following federal funding. Industry involvement in policy advocacy generated public skepticism. |
| Harmon et al., 2024 | Vaccine Procurement: The Changes Needed to Close Access Gaps and Achieve Health Equity in Routine and Pandemic Settings | General |  | Global with Canadian Relevance | Formal: Funding Mechanisms; Informal: Inter-organizational Coordination | Canada secured advance purchase agreements for over 300 million vaccine doses across seven manufacturers. Government investment focused on building domestic biomanufacturing capacity alongside international procurement. |
| Hobbs & Buxton, 2014 | Influenza immunization in Canada's low-income population. | Influenza | Low-income Canadians (n=10,373) | Canada | Formal: Funding Mechanisms; Informal: Information Access and Trust | Low-income individuals receiving social assistance or seniors' benefits were more likely to receive influenza vaccination regardless of provincial funding model, suggesting targeted outreach to high-risk groups was effective. |
| Houle & Eurich, 2019 | Completion of multiple-dose travel vaccine series and the availability of pharmacist immunizers: A retrospective analysis of administrative data in Alberta, Canada | hepatitis A monovalent, hepatitis B monovalent,  combined hepatitis A&B, Japanese encephalitis and rabies. | Patients initiating travel vaccine series (n=393,080) | Alberta | Formal: Administrative Infrastructure; Formal: Scope-of-Practice Regulation; Formal: Service Delivery Design; Informal: Professional Norms (Pharmacists) | Travel vaccine access was complicated by physician stocking practices and cold chain concerns. Authorized pharmacist immunizers in Alberta modestly increased travel vaccine series completion rates. Pharmacists reported less comfort with specialized travel vaccines. |
| Houle et al., 2022 | Identifying vaccination deserts: The availability and distribution of pharmacists with authorization to administer injections in Ontario. | "pharmacist-administered immunizations" | Community pharmacists (n=11,436) | Ontario | Formal: Scope-of-Practice Regulation; Formal: Service Delivery Design; Informal: Professional Norms (Pharmacists) | Geographic disparities in pharmacist immunization access were identified, with rural communities having lower availability. Training and novel outreach approaches were recommended for remote areas. |
| Houle et al., 2024 | Uptake and outcomes of VaxCheck, an adult life-course vaccination service: A study among community pharmacists. | General | Adults aged 18+ (n=123) | Ontario | Informal: Professional Norms (Pharmacists) | Community pharmacy vaccination programs were well received by patients. Scope-of-practice limitations and restricted access to publicly funded vaccine supply contributed to missed vaccination opportunities. |
| Huang et al., 2010 | Trends in vaccine-induced immunity to hepatitis B among Canadian street-involved youth. | Hepatitis B | Youth (n=4,035) | Canada | Formal: Program Delivery Structures; Informal: Information Access and Trust | School attendance was associated with higher hepatitis B vaccine coverage among street-involved youth. Creative outreach programs combining community-based clinics and active recall systems increased immunization rates. |
| Humble et al., 2023 | Routine childhood vaccination among ethnocultural groups in Canada during the COVID-19 pandemic: A national cross-sectional study. | Routine childhood vaccines | Parents from diverse ethnocultural groups (n=2,531) | Canada | Informal: Cultural Constraints | Vaccination inequities among newcomers were attributed to delayed healthcare coverage, language barriers, lack of culturally relevant care, and discrimination when accessing health services. |
| Huot et al., 2010 | Adult immunization services: steps have to be done. | General | Adolescents and adults; physicians and nurses interviewed (n=57) | Québec | Formal: Service Delivery Design | Adolescent and adult vaccination services through public health clinics varied in scope, with some vaccines planned for future expansion and others underoffered due to resource constraints. |
| Ilesanmi et al., 2022 | Trends, barriers and enablers to measles immunisation coverage in Saskatchewan, Canada: A mixed methods study. | Measles | Children under 2 years (n=16,582); key informants (n=18) | Saskatchewan | Formal: Service Delivery Design | Home visitation, transportation provision, and gift card incentives were identified as strategies to address clients' broader needs alongside vaccination. |
| Isenor et al., 2016 | Impact of pharmacists as immunizers on influenza vaccination coverage in the community-setting in Nova Scotia, Canada: 2013-2015. | Influenza | Population-level census and immunization data | Nova Scotia | Informal: Professional Norms (Pharmacists) | Introduction of pharmacy-based influenza vaccination in British Columbia led to sustained increases in immunization rates among older adults over two seasons. |
| Isenor et al., 2020 | Pharmacists as immunizers to Improve coverage and provider/recipient satisfaction: A prospective, Controlled Community Embedded Study with vaccineS with low coverage rates (the Improve ACCESS Study): Study summary and anticipated significance | Hepatitis A, Hepatitis B, High-dose TIV, HZ, MenACWY, MenB, Tdap, and Typhoid feve |  | New Brunswick and Nova Scotia | Formal: Funding Mechanisms; Formal: Regulatory Frameworks; Formal: Service Delivery Design; Informal: Information Access and Trust; Informal: Professional Norms (Pharmacists) | Many adult vaccines lacked public funding, adding out-of-pocket costs to patient decision-making. Pharmacist legislation for vaccine administration varied by province. Community pharmacies were valued for accessibility, particularly in rural settings. |
| Ismail et al., 2020 | Key populations for early COVID-19 immunization: Preliminary guidance for policy | COVID-19 |  | Canada | Formal: Administrative Infrastructure; Formal: Regulatory Frameworks; Formal: Service Delivery Design; Informal: Cultural Constraints; Informal: Information Access and Trust; Informal: Inter-organizational Coordination | NACI recommended ethical, equity-based COVID-19 vaccine prioritization for high-risk populations. Transparent communication was emphasized. Implementation challenges included cold chain logistics, immunization registry integration, and access barriers for remote and Indigenous communities. |
| Kadio et al., 2024 | Facilitators and challenges in collaboration between public health units and faith-based organizations to promote COVID-19 vaccine confidence in Ontario. | COVID-19 | Public health units (n=34) | Ontario | Informal: Inter-organizational Coordination | Five facilitators for public health-faith-based organization vaccine collaboration were identified: pre-existing community relationships, co-designed strategies, trusted clinic locations, addressing social determinants, and mobilizing community leaders. |
| Kandulu et al., 2024 | A Scoping Review of Factors Affecting COVID-19 Vaccination Uptake and Deployment in Global Healthcare Systems | COVID-19 |  | Global | Formal: Funding Mechanisms; Formal: Regulatory Frameworks; Formal: Service Delivery Design; Informal: Community-Based Delivery Norms; Informal: Information Access and Trust; Informal: Inter-organizational Coordination; Informal: Professional Norms (Pharmacists) | Federal government coordination with provincial stakeholders facilitated vaccine procurement and distribution. Pharmacists and midwives were effective at vaccinating large populations. Training of non-health workers, including volunteers and students, expanded deployment capacity. |
| Kaposy & Bandrauk, 2012 | Prioritizing vaccine access for vulnerable but stigmatized groups | Influenza |  | Canada | Formal: Regulatory Frameworks; Formal: Service Delivery Design; Informal: Cultural Constraints; Informal: Inter-organizational Coordination | Federal-provincial jurisdictional dynamics led to inconsistent H1N1 vaccine prioritization. PHAC recommendations were not binding, and provincial prioritization decisions diverged, with stigmatized populations (e.g., severely obese, incarcerated) inconsistently included. |
| Kaposy, 2011 | The influence of the stigma of obesity on H1N1 influenza vaccine sequencing in Canada in 2009. | Influenza | People with obesity (n=not specified) | Canada | Informal: Cultural Constraints | Despite evidence of elevated H1N1 risk, severely obese individuals were inconsistently prioritized across Canadian provinces, with only 6 of 10 including obesity on vaccine priority lists. |
| Kassianos et al., 2021 | Key policy and programmatic factors to improve influenza vaccination rates based on the experience from four high-performing countries | Influenza |  | Global | Formal: Funding Mechanisms; Formal: Health System Governance; Informal: Information Access and Trust; Informal: Inter-organizational Coordination | Forty-two key factors for successful influenza programs were identified across five pillars: health authority accountability, facilitated access, healthcare professional engagement, disease burden awareness, and vaccination benefit belief. Multi-partner mobilization was critical. |
| Kelly et al., 2023 | Characteristics of immunisation support programmes in Canada: a scoping review and environmental scan. | General | General public and health authority stakeholders (n=124); scoping review | Canada | Formal: Administrative Infrastructure; Formal: Geographic and Logistical Access; Informal: Cultural Constraints; Informal: Inter-organizational Coordination | Multidisciplinary delivery teams facilitated vaccine program implementation. Cultural and language barriers prevented timely COVID-19 vaccination among some ethnic groups. Technology access, documentation requirements, and travel challenges posed additional barriers. |
| Khan et al., 2023 | Barriers and facilitators in uptake of human papillomavirus vaccine across English Canada: A review. | HPV | 165 included studies | Canada | Formal: Health System Governance; Informal: Cultural Constraints | Vaccine access determinants operated at provider (appropriateness, affordability, availability), patient (ability to engage, pay, reach), and system (provision, catch-up programs, supply chain) levels. |
| Khan et al., 2023 | A survey of Alberta pharmacists' actions and opinions in regard to administering vaccines and medications by injection. | Influenza | Pharmacists in Alberta (n=397) | Alberta | Formal: Administrative Infrastructure; Formal: Funding Mechanisms; Formal: Health System Governance; Formal: Scope-of-Practice Regulation | Pharmacy-level barriers included cold chain storage, injection comfort, and documentation requirements. Lack of automated immunization registry reporting and unfunded vaccine costs limited pharmacy-based immunization services. |
| Kholina et al., 2022 | An equitable vaccine delivery system: Lessons from the COVID-19 vaccine rollout in Canada. | COVID-19 | Public health officials, frontline healthcare workers, union leaders, health scholars (n=34) | Alberta, Ontario, Nova Scotia, and Yukon | Formal: Service Delivery Design; Informal: Cultural Constraints; Informal: Inter-organizational Coordination | Community organizations and primary care providers drove targeted vaccine outreach with limited provincial support. Mass immunization sites created barriers for vulnerable communities; local, culturally safe, barrier-free clinics were recommended instead. |
| Kwong et al., 2008 | Impact of varicella vaccination on health care outcomes in Ontario, Canada: effect of a publicly funded program?. | Varicella | Ontario population (n=not specified; administrative health data) | Ontario | Formal: Funding Mechanisms | Introduction of publicly funded varicella vaccination in Ontario reduced hospitalizations, emergency visits, and physician visits by over 50%, with benefits extending beyond the target age group through herd protection. |
| Lam et al., 2010 | Seasonal influenza vaccination campaigns for health care personnel: systematic review. | Influenza | Healthcare personnel in long-term care, hospitals, and primary care (n=not specified) | Canada | Informal: Information Access and Trust | Education, promotion, and improved vaccine access were the most common strategies to increase healthcare personnel influenza vaccination, though no included campaign achieved recommended coverage levels. |
| Lind et al., 2015 | How rural and urban parents describe convenience in the context of school-based influenza vaccination: a qualitative study. | Influenza | Rural parents (n=48) | Alberta | Formal: Geographic and Logistical Access; Formal: Program Delivery Structures | School-based influenza vaccination reduced demands on parental resources including scheduling, travel, and lost work time. Transportation barriers differed between rural (long distances) and urban (parking, traffic) settings. |
| Lissinna et al., 2024 | A Missed Opportunity: Evaluating Immunization Status and Barriers in Hospitalized Children. | Routine childhood vaccines | Children (n=244) | Canada | Formal: Program Delivery Structures; Formal: Service Delivery Design | School-based immunization programs were limited to school-aged children. Hospital admissions and emergency visits were identified as missed vaccination opportunities, particularly for individuals without regular healthcare access. |
| Lyeo et al., 2023 | Predictors of transportation-related barriers to healthcare access in a North American suburb. | General | Adults (n=528) | Scarborough, Ontario | Formal: Geographic and Logistical Access | Public transit users were more likely to experience transportation-related barriers to healthcare access. Active travel users were more likely to decline or postpone vaccinations due to transportation challenges. |
| Lyeo et al., 2024 | Predictors of transportation-related barriers to healthcare access in a North American suburb | General | Adults (n=528) | Ontario | Formal: Geographic and Logistical Access | Active travel users were more likely to decline vaccinations due to transportation constraints. Full-time employees faced scheduling difficulties for vaccine appointments outside work hours. |
| Lyons et al., 2024 | A Qualitative Analysis of the Functions of Primary Care Nurses in COVID-19 Vaccination. | COVID-19 | Nurses (n=76) | British Columbia, Ontario, Newfoundland and Labrador, and Nova Scotia | Informal: Professional Norms (Nurses/Physicians) | Primary care nurses across four provinces administered COVID-19 vaccines, managed documentation, and staffed outreach programs including mobile units and shelter-based clinics to reduce physical access barriers for priority populations. |
| MacDonald et al., 2022 | One child, one appointment: how institutional discourses organize the work of parents and nurses in the provision of childhood vaccination for First Nations children. | "diphtheria-tetanus-pertussis-polio (DTaP-IPV) vaccine" | First Nations parents (n=33); healthcare staff (n=6) | Alberta | Formal: Service Delivery Design | A remote community operated one main health centre and three satellite centres for vaccination. No public transportation was available, though community-organized transport to appointments was provided. |
| MacDonald et al., 2024 | Patterns in COVID-19 vaccination among children aged 5-11 years in Alberta, Canada: Lessons for future vaccination campaigns. | COVID-19 | Children (n=377,103) | Alberta | Formal: Service Delivery Design | COVID-19 vaccine distribution was concentrated in public health centres (94.4%), with pharmacies (4.2%) and other providers supplementing access in areas lacking public health infrastructure. |
| MacDougall et al., 2016 | Routine immunization of adults by pharmacists: Attitudes and beliefs of the Canadian public and health care providers. | General | Canadian adults (n=4,023) and healthcare providers (n=1,167); focus groups (n=62) | Canada | Informal: Professional Norms (Pharmacists) | Moderate public and pharmacist support existed for expanded pharmacist immunization, though a substantial minority of nurses (32%) and physicians (46%) opposed expanded pharmacist vaccination roles. |
| MacKay et al., 2024 | Confidence and barriers: Analysis of factors associated with timely routine childhood vaccination in Canada during the COVID-19 pandemic. | Routine childhood vaccines | Parents (n=2,036) | Canada | Formal: Administrative Infrastructure | Difficulty obtaining appointments was the strongest predictor of missed or delayed childhood vaccinations among opportunity-related barriers. |
| MacPherson et al., 2023 | Falling Short of Clinical Recommendations: Low Uptake of the Human Papillomavirus Vaccine Among Gay, Bisexual, and Other Men Who Have Sex with Men in Ontario, Canada. Results from the Ontario Gay Men Health Survey | HPV | Men who have sex with men (n=1,960) | Ontario | Formal: Funding Mechanisms; Formal: Geographic and Logistical Access; Formal: Program Delivery Structures; Formal: Service Delivery Design; Informal: Cultural Constraints; Informal: Information Access and Trust; Informal: Inter-organizational Coordination | HPV vaccine cost (up to $550 CAD) was prohibitive for MSM over 26 despite NACI recommendations. Sexual health clinics were strongly associated with vaccine uptake. Online resources and dating apps were proposed outreach channels for rural and older MSM. |
| Malkin et al., 2022 | Factors influencing human papillomavirus school-based immunization in Alberta: A mixed-methods study protocol. | "The Gardasil® 9 vaccine is extremely immunogenic and effective." | Youth aged 11-15 and parents of children aged 11-17 | Alberta | Formal: Program Delivery Structures; Informal: Cultural Constraints; Informal: Professional Norms (Nurses/Physicians) | Logistical barriers in school-based HPV immunization included obtaining consent forms and student records. Nurses reported insufficient capacity for school immunizations alongside existing workloads. |
| Malkin et al., 2024 | Individual and Geospatial Determinants of Health Associated With School-Based Human Papillomavirus Immunization in Alberta: Population-Based Cohort Study. | HPV | Adolescents born in 2004 in Alberta (n=45,094) | Alberta | Formal: Funding Mechanisms; Formal: Program Delivery Structures; Informal: Cultural Constraints; Informal: Professional Norms (Nurses/Physicians) | Alberta's school-based HPV program expanded eligibility over time. Low health system use was associated with reduced HPV vaccine uptake, and colonization-related trauma discouraged trust in government-provided care among some Indigenous communities. |
| Manca et al., 2024 | ‘It’s really embarrassing […] to ask for help:’ navigating invisible and intersecting inequities in barriers to getting vaccinated | COVID-19 | Individuals experiencing systemic oppression (n=27) | Canada | Formal: Geographic and Logistical Access; Formal: Service Delivery Design; Informal: Cultural Constraints; Informal: Information Access and Trust; Informal: Inter-organizational Coordination | First Nations and Métis organizations led community vaccine initiatives addressing systemic government and healthcare failures. Increased vaccine availability did not equate to improved accessibility given persistent transportation, language, and informational barriers. |
| Manji et al., 2024 | Improving Influenza Vaccine Uptake During Pregnancy Through Vaccination at Point of Care: A Before-and-After Study. | Influenza | Pregnant individuals (n=8,289) | Alberta |  |  |
| Mansell et al., 2017 | Pharmacists' Scope of Practice: Supports for Canadians with Diabetes. | General | N/A | Canada | Formal: Funding Mechanisms; Informal: Professional Norms (Pharmacists) | Provincial variation in pharmacist injection authority and vaccine eligibility created interprovincial inequities. Most provinces offered universal publicly funded influenza vaccination through pharmacies. |
| Marfo et al., 2024 | Intersecting Inequities in COVID-19 Vaccination: A Discourse Analysis of Information Use and Decision-Making Among Ethnically Diverse Parents in Canada. | COVID-19 | Parents (n=48) | Canada | Formal: Regulatory Frameworks; Informal: Cultural Constraints | Essential workers without early vaccine access faced compounded disadvantage. Racialized and Indigenous parents identified historical and contemporary racism as barriers to trust in the healthcare system and vaccine access. |
| Mark Doherty & Privor-Dumm, 2024 | Role of new vaccinators/pharmacists in life-course vaccination | General |  | Global | Formal: Administrative Infrastructure; Formal: Scope-of-Practice Regulation; Formal: Service Delivery Design; Informal: Community-Based Delivery Norms; Informal: Professional Norms (Pharmacists) | Mobile vaccination units during COVID-19 expanded coverage. Pharmacists lacked methods to identify unvaccinated patients or access provincial immunization records. Staff time shortages and perceived training gaps limited pharmacy-based vaccination. Midwife involvement remained limited. |
| Mathews et al., 2023 | An analysis of policies supporting the roles of family physicians in four regions in Canada during the COVID-19 pandemic. | COVID-19 |  | British Columbia, Newfoundland and Labrador, Nova Scotia, Ontario | Formal: Health System Governance; Formal: Service Delivery Design; Informal: Professional Norms (Nurses/Physicians) | Public ownership and expenditure policies supported mass vaccination sites, virtual care, and family physician redeployment. Family physicians prioritized high-risk patients, counselled hesitant individuals, vaccinated long-term care residents, and staffed mass and mobile clinics. |
| Mathews et al., 2023 | The roles of family physicians during a pandemic. | Any. | Family physicians (n=68) | British Columbia, Newfoundland and Labrador, Nova Scotia, Ontario | Informal: Professional Norms (Nurses/Physicians) | Family physicians managed increased demand during COVID-19, moderating specialist referrals, limiting routine lab testing, and adapting to reduced in-person services, with implications for preventive care including vaccination. |
| McDonald et al., 2024 | Creating a Low-Stimulus Clinic to improve immunization success rates for children with alternate environment needs: A quality improvement initiative. | COVID-19 | Patients and caregivers at a laterality screening clinic (n=712) | Alberta | Formal: Service Delivery Design | A low-sensory vaccination clinic design with reduced stimuli, private rooms, and extended appointment times improved the vaccination experience for sensory-sensitive populations. |
| McIntosh & Safadi, 2012 | Epidemiology and prevention of meningococcal disease: A critical appraisal of vaccine policies | Meningococcal vaccine |  | Global with Canadian Relevance | Formal: Funding Mechanisms; Formal: Regulatory Frameworks; Formal: Service Delivery Design; Informal: Information Access and Trust | Cost-effectiveness analysis informed toddler meningococcal vaccination strategies with catch-up programs. Herd protection from adolescent vaccination reduced disease in unvaccinated age groups. Improved provider education and access were recommended for adolescent vaccination. |
| Mijovic et al., 2020 | Perinatal health care providers' approaches to recommending and providing pertussis vaccination in pregnancy: a qualitative study. | tetanus toxoid, reduced diphtheria toxoid and reduced acellular pertussis (Tdap) vaccine | Perinatal healthcare providers (n=44) | British Columbia, Manitoba, Ontario, Quebec and Nova Scotia | Formal: Funding Mechanisms; Formal: Service Delivery Design; Informal: Information Access and Trust | Tdap vaccine access for pregnant women varied by province, with some lacking public funding. Point-of-care vaccination at perinatal visits was effective but not feasible in all settings. Provider recommendation was highly valued by patients. |
| Moscou et al., 2024 | Broken Promises: Racism and Access to Medicines in Canada. | COVID-19 | Community members (n=27) | Ontario | Formal: Funding Mechanisms; Formal: Geographic and Logistical Access; Formal: Regulatory Frameworks; Informal: Cultural Constraints; Informal: Professional Norms (Pharmacists) | Out-of-pocket costs and lack of health insurance were barriers for low-income populations. Racism in healthcare deterred access for Black and Indigenous patients. Inadequate transportation limited childhood vaccination attendance. Pharmacy access was directly linked to vaccine availability. |
| Musto et al., 2013 | Social equity in Human Papillomavirus vaccination: a natural experiment in Calgary Canada. | HPV | Girls (n=35,592) | Alberta | Formal: Geographic and Logistical Access; Formal: Program Delivery Structures | In-school HPV vaccination delivery achieved higher uptake than community-based (public health clinic) models. Girls in the most materially deprived neighbourhoods were disproportionately disadvantaged by community delivery. |
| Nickel et al., 2024 | COVID-19 diagnostic testing and vaccinations among First Nations in Manitoba: A nations-based retrospective cohort study using linked administrative data, 2020-2021. | COVID-19 | First Nations (n=114,816) | Manitoba | Formal: Regulatory Frameworks; Informal: Inter-organizational Coordination | First Nations-led pandemic coordination ensured culturally appropriate information and priority vaccination access. Collaborative advocacy between First Nations leadership and the Manitoba government improved vaccine access for communities facing systemic barriers. |
| Okoli et al., 2024 | A population-based, province-wide, record-linkage interrupted time series analysis of impact of the universal seasonal influenza vaccination policy on seasonal influenza vaccine uptake among 5-64-year-olds in the province of Manitoba, Canada | Influenza | Manitoba residents aged 6+ months (n=25,032,069 subject-seasons) | Manitoba | Formal: Funding Mechanisms; Formal: Service Delivery Design; Informal: Professional Norms (Pharmacists) | Manitoba's transition from targeted to universal publicly funded influenza vaccination expanded access. Geographic disparities in pharmacy-based immunization availability persisted, though pharmacist immunizers modestly increased uptake overall. |
| Papastergiou et al., 2014 | Community pharmacist-administered influenza immunization improves patient access to vaccination. | Influenza | Pharmacy-immunized patients (n=1,502) | Ontario | Informal: Professional Norms (Pharmacists) | Pharmacist-administered influenza vaccination was well received by patients. Expanding pharmacist vaccination to additional vaccines was anticipated to improve public health outcomes and patient convenience. |
| Piche-Renaud et al., 2021 | Impact of the COVID-19 pandemic on the provision of routine childhood immunizations in Ontario, Canada. | HPV | Family physicians (n=189) and pediatricians (n=286) | Ontario | Formal: Administrative Infrastructure; Formal: Health System Governance | Routine immunizations were designated an essential health service during the pandemic. Proposed solutions included dedicated vaccination centres, centralized electronic immunization records, and PPE access support. |
| Piedimonte et al., 2018 | Impact of an HPV Education and Vaccination Campaign among Canadian University Students. | HPV | University students (n=151) | Quebec | Informal: Information Access and Trust | University students at increased HPV risk demonstrated poor knowledge and low perceived risk. Access to gynecological care and prior STI education were associated with increased vaccination. |
| Poudel et al., 2019 | Pharmacist role in vaccination: Evidence and challenges | General |  | Global with Canadian Relevance | Formal: Administrative Infrastructure; Formal: Funding Mechanisms; Formal: Geographic and Logistical Access; Formal: Service Delivery Design; Informal: Information Access and Trust; Informal: Inter-organizational Coordination; Informal: Professional Norms (Pharmacists) | Pharmacy-based vaccination increased uptake but excluded publicly funded vaccines in some jurisdictions. Missed vaccination opportunities, absence of population-based reminder systems, and patient out-of-pocket costs were persistent barriers. Pharmacists were recognized as accessible, effective immunizers. |
| Powis et al., 2022 | Factors associated with timely receipt of COVID vaccination in patients with cancer | COVID-19 | Cancer patients (n=356,535) | Ontario | Formal: Geographic and Logistical Access; Formal: Regulatory Frameworks; Formal: Service Delivery Design; Informal: Cultural Constraints; Informal: Information Access and Trust; Informal: Inter-organizational Coordination | Community partnerships using participatory design improved COVID-19 vaccination in racialized populations. Mobile clinics and representative healthcare providers were effective strategies. Systemic barriers including transportation and health system navigation persisted. |
| Powis et al., 2023 | Factors associated with timely COVID-19 vaccination in a population-based cohort of patients with cancer. | COVID-19 | Cancer patients (n=356,535) | Ontario | Formal: Funding Mechanisms; Formal: Health System Governance; Informal: Cultural Constraints | Ontario prioritized high-risk groups including cancer patients, Indigenous populations, and high-exposure neighbourhoods for COVID-19 vaccination. Despite universal funding, marginalized populations remained less likely to be vaccinated due to systemic barriers. |
| Rambout et al., 2014 | Self-reported barriers and facilitators to preventive human papillomavirus vaccination among adolescent girls and young women: A systematic review | HPV | Females aged 9-26 (n=8,079); 22 included studies | Global with Canadian Relevance | Formal: Funding Mechanisms; Formal: Geographic and Logistical Access; Formal: Service Delivery Design; Informal: Information Access and Trust | Vaccine cost was a major barrier, intensifying with higher prices. Transportation (22%) and lack of vaccine knowledge (31-40%) were frequently reported barriers. Many unvaccinated individuals had not returned to clinic or were not offered the vaccine when they did. |
| Ranjan et al., 2019 | Barriers and facilitators to hepatitis B vaccination among sex workers in Vancouver, Canada: Implications for integrated HIV, STI, and viral hepatitis services | hepatitis B | Sex workers (n=855) | British Columbia | Formal: Program Delivery Structures; Formal: Regulatory Frameworks; Formal: Service Delivery Design; Informal: Community-Based Delivery Norms; Informal: Cultural Constraints; Informal: Inter-organizational Coordination | School-based HBV vaccination and integrated harm reduction programs facilitated vaccine access for marginalized sex workers. Criminalization, stigma, and unsafe working conditions reduced healthcare disclosure and vaccination eligibility. |
| Ricciardi et al., 2015 | Comparison of NITAG policies and working processes in selected developed countries | General |  | Global with Canadian Relevance | Formal: Regulatory Frameworks; Informal: Information Access and Trust | Transparent decision-analysis frameworks were critical for public confidence in national immunization programs, though restricted access to meeting agendas, minutes, and reports limited accountability. |
| Robitaille et al., 2023 | Community Pharmacists and Influenza Vaccination: Opportunities and Challenges From a Public Health Perspective. | Influenza | Pharmacists, pharmacy nurses, association and CISSS representatives (n=23) | Quebec | Formal: Health System Governance; Informal: Inter-organizational Coordination; Informal: Professional Norms (Pharmacists) | First-come-first-served vaccine distribution raised equity concerns. Inter-institutional coordination was insufficient. Quebec legislation extended vaccination authority to pharmacists, improving access but introducing financial sustainability challenges. |
| Romain & Schillaci, 2009 | Childhood immunization: Availability of primary care providers in Ontario. | Measles | Children; population-level immunization coverage data (2000-2004) | Ontario | Formal: Service Delivery Design; Informal: Professional Norms (Nurses/Physicians) | Childhood immunization coverage fluctuations correlated with changes in the relative number of family physicians and pediatricians, suggesting primary care provider supply influenced vaccination rates. |
| Rubens-Augustson et al., 2019 | Healthcare provider perspectives on the uptake of the human papillomavirus vaccine among newcomers to Canada: a qualitative study. | Human papillomavirus vaccine | Newcomers; healthcare providers (n=10) | Ontario | Formal: Funding Mechanisms; Informal: Information Access and Trust | HPV vaccination among newcomers was influenced by interrelated patient, provider, and system-level factors. Providers identified systemic gaps including insufficient time and resources for vaccine promotion. |
| Russell et al., 2005 | Improving the public health/physician partnership for influenza vaccination. | Influenza | Family physicians/general practitioners (n=1,243) | Alberta | Informal: Professional Norms (Nurses/Physicians) | Most Alberta family physicians provided influenza vaccination; those using non-public-health vaccine suppliers were more likely to charge patients, creating potential financial access barriers. |
| Santangelo et al., 2024 | COVID-19 Vaccination and Public Health: Addressing Global, Regional, and Within-Country Inequalities | COVID-19 |  | Global | Formal: Regulatory Frameworks; Formal: Service Delivery Design; Informal: Information Access and Trust; Informal: Inter-organizational Coordination | Vaccine task forces and community ambassadors facilitated engagement with equity-deserving groups. Mobile vaccination units reduced logistical barriers by bringing services directly to underserved communities. Culturally relevant messaging improved acceptance. |
| Sauvageau et al., 2013 | Immunization services offered in Quebec (Canada) pharmacies. | General | Pharmacists (n=1,102) | Québec | Informal: Professional Norms (Pharmacists) | Half of Quebec pharmacists surveyed would administer vaccines if legislation permitted. High pharmacist interest in expanded vaccination services was identified, with potential to complement physician-based immunization. |
| Scheifele et al., 2014 | Approved but non-funded vaccines: accessing individual protection. | Recommended, but unfunded vaccines | Not specified | Canada | Informal: Professional Norms (Nurses/Physicians); Informal: Professional Norms (Pharmacists) | Growing pharmacy authorization to dispense and administer recommended-but-unfunded vaccines increased access. Physician liability concerns regarding unvaccinated patients prompted increased discussion of all recommended vaccines. |
| Scott & Batty, 2016 | HPV Vaccine Uptake Among Canadian Youth and The Role of the Nurse Practitioner. | HPV | N/A | Canada | Informal: Information Access and Trust; Informal: Professional Norms (Nurses/Physicians) | Lack of HPV disease and vaccine information was a key barrier for families. Nurse practitioner recommendation was a significant facilitator of vaccine uptake. |
| Sebring et al., 2022 | "None of it was especially easy": improving COVID-19 vaccine equity for people with disabilities. | COVID-19 | Manitobans with disabilities (n=23) | Manitoba | Formal: Administrative Infrastructure; Informal: Information Access and Trust | Three routine barrier areas for COVID-19 vaccine access were identified: information and appointment booking, physical access to vaccination clinics, and the vaccination experience itself. |
| Sibley & Weiner, 2011 | An evaluation of access to health care services along the rural-urban continuum in Canada. | General | Adults (n=111,258) | Canada | Formal: Funding Mechanisms; Informal: Professional Norms (Nurses/Physicians) | Interprovincial variation in influenza vaccination was linked to differences in publicly funded programs, with Ontario's universal program associated with highest uptake. Rural-urban disparities in specialist access persisted despite equitable family physician utilization. |
| Simms et al., 2023 | COVID-19 vaccine behaviour among citizens of the Metis Nation of Ontario: A qualitative study. | COVID-19 | Métis citizens (n=16) | Ontario | Formal: Administrative Infrastructure; Formal: Health System Governance; Formal: Regulatory Frameworks; Informal: Information Access and Trust | Lack of domestic vaccine production posed structural barriers. Métis communities valued prioritization but some experienced guilt over perceived inequity. Online booking systems and inconsistent government messaging created additional access barriers. |
| Song et al., 2024 | Public health partnerships with faith-based organizations to support vaccination uptake among minoritized communities: A scoping review | General |  | Global | Formal: Geographic and Logistical Access; Formal: Service Delivery Design; Informal: Cultural Constraints; Informal: Information Access and Trust; Informal: Inter-organizational Coordination | Public health-faith-based organization coalitions provided transportation, social services, and wrap-around supports for vaccine seekers. Faith-based organizations facilitated access in rural and remote communities where public health agencies had limited reach. |
| Spinks et al., 2020 | Does policy change to allow pharmacist provision of influenza vaccination increase population uptake? A systematic review | Influenza | N/A | Global | Informal: Professional Norms (Pharmacists) | Canadian provinces with pharmacist vaccination policies had higher influenza vaccination rates. Pharmacy-based vaccination attracted first-time and occasional vaccine recipients, complementing traditional healthcare settings. |
| Stratoberdha et al., 2022 | Barriers to adult vaccination in Canada: A qualitative systematic review. | HPV | 34 included studies; general population, Indigenous peoples, immigrants, healthcare professionals | Canada | Informal: Information Access and Trust; Informal: Professional Norms (Pharmacists) | Lack of vaccine information (41%) and unawareness of vaccine existence (29%) were the most frequently reported barriers. Pharmacists were positioned to address hesitancy through education and administration. |
| Thambinathan et al., 2024 | "Access to healthcare is a human right": a constructivist study exploring the impact and potential of a hospital-community partnered COVID-19 community response team for Toronto homeless services and congregate living settings. | COVID-19 | Staff (n=10) | Toronto, Ontario | Formal: Administrative Infrastructure; Formal: Health System Governance; Formal: Service Delivery Design; Informal: Cultural Constraints; Informal: Inter-organizational Coordination | Inconsistent vaccine stocking across clinics created access barriers. A hospital-shelter partnership improved vaccine delivery to homeless populations, though processes were considered administratively burdensome. |
| Thidrickson & Goodyer, 2019 | Pharmacy Travel Health Services in Canada: Experience of Early Adopters. | General | Pharmacists (n=21) | British Columbia, Alberta, Manitoba, Ontario, New Brunswick, Nova Scotia, Prince Edward Island | Informal: Professional Norms (Pharmacists) | Scope-of-practice restrictions, including lack of prescribing authority for antimalarials and limited travel vaccine authorization, hindered pharmacist-led travel health services across provinces. |
| Tinessia et al., 2024 | Strategies to address COVID-19 vaccine hesitancy in First Nations peoples: a systematic review. | COVID-19 | 17 included studies | Global | Formal: Service Delivery Design; Informal: Cultural Constraints; Informal: Inter-organizational Coordination | Community-controlled, culturally safe vaccination clinics led by Indigenous health professionals facilitated trust and uptake. Vaccine confidence among Indigenous peoples was linked to historical colonial violence and ongoing distrust. |
| Turner et al., 2015 | Barriers to Influenza Vaccination in Patients with Implantable Cardiac Defibrillators. | Influenza | Patients with implantable cardiac defibrillators (n=229) | Ontario | Informal: Information Access and Trust | Patient beliefs about influenza vaccine safety and efficacy were the only independent predictor of vaccine receipt among implantable cardiac device patients, in a universal healthcare context where cost was not a barrier. |
| Valaitis et al., 2020 | Addressing quadruple aims through primary care and public health collaboration: ten Canadian case studies. | General | Healthcare providers (n=328) | Nova Scotia, Ontario, and British Columbia | Formal: Service Delivery Design; Informal: Information Access and Trust | Primary care and public health collaboration leveraged pre-established community relationships to deliver services to hard-to-reach populations, supporting both patient experience and population health goals. |
| Vanderslott & Marks, 2021 | Charting mandatory childhood vaccination policies worldwide | Measles |  | Global | Formal: Program Delivery Structures; Formal: Regulatory Frameworks; Informal: Inter-organizational Coordination | Mandatory vaccination policies were most commonly linked to school or childcare entry. Subnational variation in governance arrangements, local politics, and vaccination attitudes created within-country differences in implementation. |
| Vernon-Wilson et al., 2023 | Unintended consequences of communicating rapid COVID-19 vaccine policy changes- a qualitative study of health policy communication in Ontario, Canada. | COVID-19 | Policy communicators and community leaders (n=29) | Ontario | Informal: Information Access and Trust | Rapidly changing COVID-19 vaccine policy created communication barriers. Multi-modal dissemination was a strength, but frequent policy updates generated confusion and undermined public confidence. |
| Vernon-Wilson et al., 2024 | Implementation of an adult life-course vaccine review service, VaxCheck, in community pharmacy: A qualitative analysis. | General | Pharmacists (n=26) | Ontario | Formal: Administrative Infrastructure; Informal: Professional Norms (Pharmacists) | A clinical decision support tool (VaxCheck) was developed for community pharmacy vaccine reviews. Pharmacists were valued as accessible providers with perceived equitable power dynamics, though workload and workflow integration posed challenges. |
| Waite et al., 2019 | Characteristics of patients vaccinated against influenza in physician offices versus pharmacies and predictors of vaccination location: a cross-sectional study. | Influenza | Influenza vaccine recipients in Ontario (n=2,677,287 and 2,465,178) | Ontario | Informal: Professional Norms (Nurses/Physicians); Informal: Professional Norms (Pharmacists) | Influenza vaccination shifted from physician offices to pharmacies over time. Loyalty, familiarity, and convenience drove patient choice of vaccination provider. |
| Waite et al., 2024 | Willingness of Canadian community pharmacists to adopt a proactive life-course approach to vaccination services. | General | Pharmacists (n=24) | Canada | Formal: Funding Mechanisms; Formal: Regulatory Frameworks; Informal: Professional Norms (Pharmacists) | Varied vaccine funding models created complexity for pharmacist-led vaccination. Rapidly changing eligibility guidelines and inadequate staffing limited proactive vaccination approaches in pharmacy settings. |
| Warmels et al., 2017 | Improving team-based care for children: shared well child care involving family practice nurses. | Vaccines for infants and children | Parents of young children (n=20) | Canada | Informal: Professional Norms (Nurses/Physicians) | A nurse-provided well-child care pilot in family practice demonstrated feasibility, shifting routine immunization and developmental assessment tasks from physicians to registered nurses. |
| Whang et al., 2023 | Implementing older adult vaccination within the Immunization Agenda 2030: A discussion of potential challenges and solutions | General |  | Global | Formal: Funding Mechanisms; Formal: Regulatory Frameworks; Formal: Service Delivery Design; Informal: Inter-organizational Coordination | Canada lacked a nationally standardized vaccination schedule, with interprovincial variation in publicly funded programs. Mobile clinics, workplace vaccination, and private-public partnerships were recommended strategies. |
| Whop et al., 2021 | Achieving cervical cancer elimination among Indigenous women | HPV |  | Global | Formal: Administrative Infrastructure | Accurate collection and reporting of Indigenous status data required staff training, investment in information systems, and Indigenous leadership in governance. |
| Yee et al., 2024 | Effectiveness, acceptability, and potential of lay student vaccinators to improve vaccine delivery. | COVID-19 | Students (n=157) | Ontario | Informal: Community-Based Delivery Norms | Lay student vaccinators with minimal training safely administered vaccines under medical directive, demonstrating potential to address healthcare worker shortages in vaccine delivery. |
| Zelman et al., 2014 | Implementation of a universal rotavirus vaccination program: comparison of two delivery systems. | Rotavirus | Infants (n=not specified); key informants (n=8) | Prince Edward Island, Nova Scotia and New Brunswick | Informal: Professional Norms (Nurses/Physicians) | Public health nurse-delivered rotavirus vaccination in PEI achieved over 90% uptake rapidly, while physician office-based delivery in Nova Scotia was hampered by low provider and caregiver awareness despite extensive outreach. |
| Zibrik et al., 2018 | Let's Talk About B: Barriers to Hepatitis B Screening and Vaccination Among Asian and South Asian Immigrants in British Columbia. | Hepatitis B | Korean, Chinese, Filipino, and Punjabi immigrants (n=827) | British Columbia | Informal: Cultural Constraints; Informal: Information Access and Trust | Immigrants faced economic, linguistic, and systemic barriers to accessing hepatitis B vaccination. Lack of awareness of HBV, its transmission, and vaccine availability was widespread across population groups. |
